# A comprehensive assessment of the existing landscape of personalized cancer medicine in the European Union, on behalf of the PCM4EU consortium

**DOI:** 10.1016/j.esmoop.2025.105872

**Published:** 2025-11-06

**Authors:** S.F. Haj Mohammad, H. van der Pol, E. Vrdoljak, U. Lassen, K. Ojamaa, K. Jalkanen, L. Verlingue, A. Stenzinger, A. Patócs, G. Curigliano, E. Baltruškevičienė, Å. Helland, H.G. Russnes, I. Ługowska, B. Mainoli, A. Edsjö, E. Lonardi, K. Taskén, H. Gelderblom

**Affiliations:** 1 Department of Medical Oncology, Leiden University Medical Center, Leiden, The Netherlands; 2 Mathematical Institute, Leiden University, Leiden, The Netherlands; 3 Department of Oncology, University Hospital Center Split, School of Medicine, Split, Croatia; 4 Department of Medical Oncology, Rigshospitalet, Copenhagen, Denmark; 5 Clinic of Hematology and Oncology, Tartu University Hospital, Tartu, Estonia; 6 Clinical Trial Unit, Helsinki University Hospital, Comprehensive Cancer Center, Helsinki, Finland; 7 Phase 1 Clinical Unit, Centre Léon Bérard, Lyon, France; 8 Institute of Pathology, Heidelberg University Hospital, Heidelberg, Germany; 9 Department of Molecular Genetics, National Institute of Oncology, National Tumor Biology Laboratory, HUN-REN-ONKOL-TTK-HCEMM Oncogenomics Research Group, National Institute of Oncology, Budapest, Hungary; 10 European Institute of Oncology, IRCCS, Milan, Italy; 11 Department of Oncology and Hematology-Oncology, University of Milan, Milan, Italy; 12 Clinical Trial Center, National Cancer Institute, Vilnius, Lithuania; 13 Institute for Cancer Research and Division of Cancer Medicine, Oslo University Hospital, Oslo, Norway; 14 Institute of Clinical Medicine, University of Oslo, Oslo, Norway; 15 Department of Pathology, Oslo University Hospital, Oslo, Norway; 16 Early Phase Clinical Trials Unit, Maria Skłodowska Curie National Research Institute of Oncology, Warsaw, Poland; 17 Clinical Research Unit, Research Center of IPO Porto (CI-IPOP)/CI-IPOP@RISE (Health Research Network), Portuguese Oncology Institute of Porto (IPO Porto)/Porto Comprehensive Cancer Center Raquel Seruca (Porto.CCC Raquel Seruca), Porto, Portugal; 18 Department of Clinical Genetics, Pathology, and Molecular Diagnostics, Skåne University Hospital, Region Skåne, Lund, Sweden; 19 Department of Research and Valorization, Leiden University Medical Center, Leiden, The Netherlands

**Keywords:** personalized cancer medicine, molecular diagnostics, biomarkers

## Abstract

**Background:**

In the growing field of personalized cancer medicine (PCM), successful implementation requires access to advanced molecular diagnostics and treatments. The Personalized Cancer Medicine for all EU Citizens (PCM4EU) consortium was established to facilitate broad implementation of PCM across Europe. This study aimed to assess the current status of PCM from the perspectives of health care professionals and patients.

**Materials and methods:**

Three distinct questionnaires were developed for medical oncologists, pathologists, and patients, focusing on molecular diagnostics use in countries participating in the PCM4EU consortium. Adult patients with locally advanced or metastatic cancer who underwent molecular diagnostics were eligible to complete the patient questionnaire.

**Results:**

Between July 2024 and February 2025, 14 out of 15 countries completed the medical oncologist and pathologist questionnaires. Equitable access to molecular diagnostics was reported by 4 out of 14 countries, with limited access to treatments, clinical trials, or reimbursement issues identified as common barriers. Tumor-specific biomarkers matching approved targeted therapies were more often tested than tumor-agnostic biomarkers. Molecular tumor boards were established in 13 out of 14 countries. There was limited availability of complex biomarker testing techniques in several countries, and laboratories sometimes lacked accreditation by the International Organization for Standardization. A total of 288 patients from 16 countries completed the patient questionnaire. More comprehensible information regarding molecular diagnostics and better pre- and post-test genetic counseling were the main improvements reported by patients.

**Conclusions:**

By use of extensive questionnaires, we assessed the implementation of PCM in Europe and identified persistent inequalities, ranging from disparities in available biomarker testing techniques to their reimbursement, and shortcomings in communication to patients. By facilitating access to similar treatments across countries with the establishment of a Drug Rediscovery Protocol-like clinical trials network, and accelerating data generation by sharing data, patients in Europe are one step closer to equitable access to precision oncology.

## Highlights

• There are persistent inequalities in access to molecular diagnostics between European countries.
• Tumor-specific biomarkers matching approved targeted therapies are more frequently tested than tumor-agnostic biomarkers.
• Patients highlight the need for more understandable information regarding molecular diagnostics and genetic counseling.

## Introduction

With nearly 3.5 million new cancer diagnoses and >1.6 million cancer-related deaths annually in Europe, the disease has a severe impact on both morbidity and mortality.^1^ The field of personalized cancer medicine (PCM) has expanded significantly over the past decades, from the development of genomic testing techniques to targeted therapies, and has become indispensable in current cancer care. A personalized-based approach, where patients get treatment based on molecular alterations in their tumors, has shown promising results regarding treatment efficacy, and at the same time reduces adverse events, enhancing quality of life.^2^^,^^3^

For successful implementation of PCM, access to advanced molecular diagnostics, treatments, and clinical trials is fundamental. Despite rapid developments, there is unequal access to PCM across and within countries in the European Union (EU).^4^ Europe’s Beating Cancer Plan aims to tackle these inequalities on all aspects of PCM. As a part of this plan, the Personalized Cancer Medicine for all EU Citizens (PCM4EU) consortium was established in 2023, co-funded by the EU4Health programme (grant: 101079984), comprising 17 different partners, including stakeholders within medical oncology, pathology, patient advocacy, and decision making, in 15 European countries, with the aim of improving survival and quality of life of patients with cancer across the EU by broadly implementing PCM.^5^

At the center of the PCM4EU consortium’s strategy is the Drug Rediscovery Protocol (DRUP), a successful nationwide PCM trial conducted in the Netherlands.^6^ Building on its success, DRUP-like clinical trials have been established across Europe, each an independent, investigator-initiated study with aligned protocols based on DRUP, forming a network of PCM trials. Achieving equitable access for all patients is a key objective of the PCM4EU consortium. However, there are knowledge gaps concerning the use of molecular diagnostics, available infrastructure, and implementation of PCM in the EU. Therefore, this study aimed to evaluate the existing landscape of PCM in European countries, covering various aspects from available biomarkers and molecular testing techniques to reimbursement and patient preferences.

## Materials and methods

### Questionnaires’ design

Three distinct questionnaires were developed between May and August 2023 for medical oncologists, pathologists (or molecular biologists), and patients. Since no validated questionnaires were available for the aim of this study, the questionnaires were designed according to the literature, as detailed for each questionnaire below, and adjusted after expert review within the PCM4EU consortium.

The questionnaire for medical oncologists focused on the availability and reimbursement of various biomarker analyses in 15 different tumor types. The tumor types and associated biomarkers were based on the 13 Dutch lists of ‘Clinically Necessary (Molecular) Targets’ (CNT lists) per tumor type that were available at the time of questionnaire development [i.e. non-small-cell lung cancer (NSCLC), colorectal cancer (CRC), pancreatic cancer, breast cancer, prostate cancer, melanoma, ovarian cancer, endometrial cancer, cervical cancer, renal cell/urothelial cancer, biliary tract cancer, gastrointestinal stromal tumor, and thyroid cancer], accompanied with glioblastoma and cancer of unknown primary (CUP) to have a representative list of tumors.^7^^,^^8^ Furthermore, the questionnaire focused on possible barriers for implementing and reasons to carry out molecular diagnostics, inspired by a questionnaire that was conducted among medical oncologists in the United States in 2017.^9^

The pathologist questionnaire concentrated on the availability and reimbursement of different biomarker testing techniques for the above-mentioned tumor types from the medical oncologist questionnaire, based on a European Society for Medical Oncology (ESMO) study from 2023 regarding the availability of biomolecular technologies across Europe.^10^ The assessed testing techniques were as follows: immunohistochemistry (IHC), FISH, PCR, microsatellite instability (MSI), small (<50 genes) and large (>50 genes) panel next-generation sequencing (NGS), targeted and full RNA sequencing (RNAseq), whole-exome sequencing (WES), whole-genome sequencing (WGS), genomic assays, liquid biopsies, and tumor mutational burden (TMB).

The questionnaire for patients was influenced by previously conducted research^11^^,^^12^ and included questions regarding their experience with molecular diagnostics and knowledge on the topic. Patients were eligible for participation if they were diagnosed with locally advanced or metastatic cancer and had undergone molecular testing. Each questionnaire was translated to the native language of the participating countries, thus resulting in 16 versions: 1 original version in English and 15 translations.

### Study design

One medical oncologist and one pathologist per participating country in the PCM4EU consortium were asked to fill in the questionnaire. These respondents were all affiliated with their ongoing DRUP-like clinical trial or were in the process of starting a similar trial within the project. Via the secured, web-based system Castor Electronic Data Capture (EDC), a personal link was sent to the dedicated participants. The patient questionnaire was disseminated through national patient organizations in the respective countries. They were provided with standardized recruitment material, including a general access link directing patients to the questionnaire in Castor EDC, which captured the data anonymously. Each time the general link was used, a new participant was generated in the database. Data were only stored upon full completion of the questionnaire by the patient.

### Statistical analysis

All statistical analyses were carried out using R version 4.4.0 (https://www.R-project.org). Descriptive statistics were used to summarize questionnaire responses. Categorical data were presented as numbers and percentages.

### Ethical considerations

The Medical Research Ethics Committee of the Leiden University Medical Center confirmed that the Medical Research Involving Human Subjects Act (Dutch abbreviation: WMO) did not apply to the study (reference number: nWMODIV2_20240620).

## Results

### Medical oncologist questionnaire

Between August and December 2024, 14 out of 15 countries (93.3%) participating in the PCM4EU project completed the medical oncologist questionnaire (Supplementary Figure S1 and Supplementary Appendix S1, available at https://doi.org/10.1016/j.esmoop.2025.105872). Molecular diagnostics was a mandatory part of the curriculum for the medical oncology specialization in 28.6% of the countries, namely Denmark, France, Hungary, and Sweden. In the other 71.4% (*n* = 10), the subject was addressed through lectures, seminars, symposia, or dedicated courses, but these activities were not mandatory.

**Figure 1:**
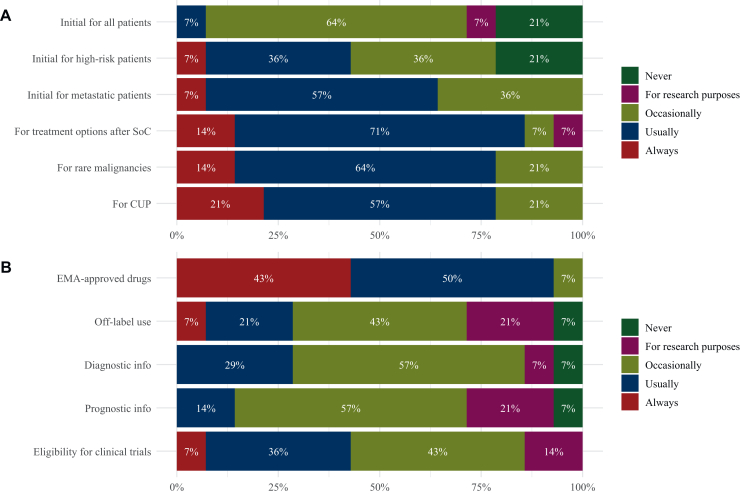
**Use of molecular diagnostics**. Bar plots show grouped answers from all countries when (A) and for what reason (B) molecular diagnostics would be used. CUP, cancer of unknown primary; EMA, European Medicines Agency. SoC, standard of care.

The use of molecular diagnostics varied depending on the stage of the cancer trajectory (Figure 1A). At the time of initial diagnosis, 28.6% of countries (*n* = 4) reported that molecular diagnostics for all patients with cancer was never carried out, or only in a research setting. The remaining countries indicated occasional or usual use for this group. However, in the metastatic setting, molecular diagnostics was either carried out occasionally (i.e. restricted use; 35.7%, *n* = 5), usually (57.1%, *n* = 8), or always (7.1%, *n* = 1). For rare malignancies and CUP, testing was carried out usually or always in 78.6% (*n* = 11) of the countries. An overview of all answers per country can be found in Supplementary Table S1, available at https://doi.org/10.1016/j.esmoop.2025.105872.

How often molecular diagnostics was carried out also depended on their intended reason (Figure 1B and Supplementary Table S1, available at https://doi.org/10.1016/j.esmoop.2025.105872). For the selection of patients eligible to receive drugs approved by the European Medicines Agency (EMA), 92.3% of the countries (*n* = 13) reported using molecular diagnostics either usually or always. In contrast, for off-label use of these drugs, only 28.6% (*n* = 4) reported such frequent use. For gathering diagnostic or prognostic information about a patient’s tumor, more than half of the countries (57.1%, *n* = 8) reported occasional use of molecular diagnostics. Regarding patient eligibility for clinical trials, six countries (42.9%) reported that molecular testing was carried out usually or always, while another six countries (42.9%) indicated occasional use, and two countries (14.3%) conducted testing only for research purposes.

**Figure 2:**
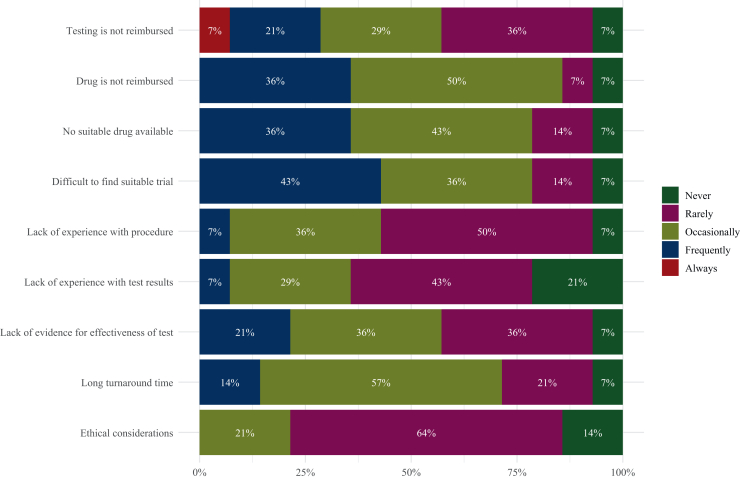
**Barriers to carrying out molecular diagnostics.** Bar plot shows grouped answers of all countries regarding potential barriers to carrying out molecular diagnostics.

With respect to barriers to carrying out molecular diagnostics, as shown in Figure 2, the absence of reimbursement for the targeted therapy corresponding to the identifiable biomarker was stated as the most significant barrier. This was reported as an occasional or frequent barrier by nearly all countries (85.7%, *n* = 12). The unavailability of a suitable drug and difficulty to find a clinical trial were the second and third most frequently reported barriers, both reported as occasional or frequent by 11 countries (78.6%). In contrast, the least frequently reported barrier was ethical considerations, which was rarely or never identified as a barrier by 78.6% of the countries (*n* = 11). Supplementary Table S2, available at https://doi.org/10.1016/j.esmoop.2025.105872, provides an overview of all answers per country. Supplementary Table S3, available at https://doi.org/10.1016/j.esmoop.2025.105872, contains additional results regarding potential barriers for reimbursement.

Supplementary Figure S2, available at https://doi.org/10.1016/j.esmoop.2025.105872, presents the reported use of different biomarkers across 15 tumor types, based on the CNT lists. While usage patterns varied across countries, certain biomarkers, such as programmed death-ligand 1 expression, *EGFR* mutations, and *ALK* fusions in NSCLC, *KRAS* and *NRAS* mutations in CRC, estrogen receptor/progesterone receptor status in breast and endometrial cancer, *BRAF*^*V600*^ mutations in melanoma, monoallelic *BRCA1/2* mutations in ovarian cancer, and *IDH* mutations in glioblastoma, were reportedly always or usually utilized. In contrast, usage of the biomarkers MSI and TMB was often reported occasionally or for research purposes, except for MSI in colorectal and endometrial cancer, which was more often carried out. However, many countries stated that both MSI and TMB are part of large NGS panels and are therefore reported anyway.

Regarding the reimbursement of molecular diagnostics, 50.0% of the countries (*n* = 7, Croatia, Denmark, Estonia, Hungary, Norway, Portugal, and Sweden) reported that testing is fully covered by public reimbursement. In France and the Netherlands, if the patient's insurance does not fully cover the costs, additional funding sources, such as public reimbursement or hospital budgets, would cover the remaining costs. Conversely, in Lithuania and Poland, patients are responsible for the remaining unreimbursed costs. In Finland and Italy, reimbursement differs a lot between hospitals. Lastly, coverage of molecular diagnostics in Germany depends on the patient’s insurance, with funding either by public or private insurance, or paid for by the patient.

Concerning equal availability and accessibility of molecular diagnostics for all patients, only four countries (28.6%, Estonia, the Netherlands, Norway, and Sweden) reported that such equality was achieved. The remaining countries indicated the presence of regional and institutional differences. Lithuania noted the main problem to be funding since most of the molecular diagnostics and respective treatments are not reimbursed. Notably, changes in reimbursement policies have been implemented in Lithuania since the completion of the questionnaire. As of February 2025, certain gene panels are now reimbursed.

### Pathologist questionnaire

The same countries that completed the medical oncologist questionnaire finished the pathologist questionnaire between July 2024 and February 2025 (Supplementary Figure S1 and Supplementary Appendix S2, available at https://doi.org/10.1016/j.esmoop.2025.105872). All countries besides Poland (*n* = 13, 92.3%) indicated the presence of a molecular tumor board (MTB) in their country. The organization of MTBs varied across countries, with several countries reporting MTBs at multiple organizational levels. Nine countries (64.3%; Croatia, Denmark, Estonia, Finland, France, Germany, Hungary, Norway, and Sweden) reported the presence of a national MTB. Regional MTBs were conducted in seven countries (50%; France, Germany, Hungary, Italy, the Netherlands, Norway, and Sweden), while nine countries (64.3%; Denmark, Finland, France, Germany, Lithuania, the Netherlands, Norway, Portugal, and Sweden) mentioned the presence of institutional MTBs. Poland stated that they are in the process of starting an MTB.

The availability of laboratories providing the different techniques varied depending on the complexity of the technique. Supplementary Figure S3, available at https://doi.org/10.1016/j.esmoop.2025.105872, shows the responses per testing technique from all countries combined. Less complex techniques, including IHC, FISH, PCR, MSI, and small NGS panels, were all carried out at public or both public and private laboratories. In contrast, more complex techniques, such as full RNAseq, WES, and WGS, were not available in 21.4% of the countries (*n* = 3; Croatia, Estonia, and Lithuania). Croatia, Denmark, Norway, and the Netherlands reported all of their available biomarker testing techniques to be carried out at public laboratories. However, in Norway, IHC can also be carried out at private hospitals. In France, Germany, Italy, and Poland (28.6%), both public and private laboratories carried out all the techniques. Notably, Estonia, Hungary, and Lithuania indicated their more advanced testing techniques, if available, to be exclusively carried out in private laboratories. An overview of the answers per country can be found in Supplementary Table S4, available at https://doi.org/10.1016/j.esmoop.2025.105872. The number of laboratories that carried out molecular diagnostics per country, along with the proportion holding accreditation by the International Organization for Standardization (ISO), is displayed in Table 1. Although the absolute numbers differed between countries, when adjusting for population size,^13^ an approximate ratio of one laboratory per 1 million residents could be seen. However, in Denmark, Estonia, Germany, and Norway, a ratio of two to three laboratories per 1 million residents was observed.

**Table 1:** Laboratories that carry out molecular diagnostics

	Laboratories (*n*)	ISO accreditation, *n* (%)	Population in 2024 (in millions)	Laboratories per million residents (*n*)
Croatia	5	0 (0)	3.9	1.3
Denmark	20	20 (100)	6.0	3.4
Estonia	4	4 (100)	1.4	2.9
Finland	7	5 (71)	5.6	1.3
France	50^a^	25^a^ (50)	68.5	0.7
Germany	150^a^	90^a^ (60)	83.5	1.8
Hungary	6	6 (100)	9.6	0.6
Italy	70	30 (43)	59.0	1.2
Lithuania	3	1 (33)	2.9	1.0
The Netherlands	15	15 (100)	17.9	0.8
Norway	17	11 (67)	5.6	3.1
Poland	23	4 (17)	36.6	0.6
Portugal	10^a^^,^^b^	N/A	10.6	0.9
Sweden	9	9 (100)	10.6	0.9

ISO, International Organization for Standardization.

a The number is an estimation as no complete overview is available.

b No precise mapping of these facilities nationwide.

The pooled availability and use of the different biomarker testing techniques per country are displayed in Table 2. All countries reported IHC to be used always or usually for all tumor types. When looking at FISH, Croatia almost never carried out this technique, while the Netherlands never used PCR for these indications. NGS panels, either small or large, were however used in a restricted way (occasionally), usually or always in all countries. The use of more complex testing techniques, such as RNAseq, WES, and WGS, was more often restricted to research purposes, if even available at all.

**Table 2:** Combined use of biomarker testing techniques

	IHC	FISH	PCR	MSI	NGS panel (small)	NGS panel (large)	RNAseq (targeted)	RNAseq (full)	WES	WGS	Genomic assay	Liquid biopsy	TMB
Croatia	Always	Never	For research purposes	Usually	Usually	Occasionally	Never	Not available	Not available	Not available	Occasionally	Occasionally	Occasionally
Denmark	Always	Occasionally	Occasionally	Occasionally	Occasionally	Occasionally	Occasionally	Occasionally	For research purposes	Occasionally	For research purposes	Occasionally	Occasionally
Estonia	Always	Usually	Usually	Usually	Never	Usually	Occasionally	Not available	Not available	Not available	Occasionally	Occasionally	Usually
Finland	Always	Occasionally	Occasionally	Occasionally	Occasionally	Occasionally	For research purposes	For research purposes	For research purposes	For research purposes	For research purposes	Occasionally	Occasionally
France	Always	Occasionally	Occasionally	Usually	Usually	Occasionally	Occasionally	Occasionally	Occasionally	Occasionally	For research purposes	Occasionally	Occasionally
Germany	Usually	Usually	Usually	Usually	Usually	Occasionally	Occasionally	For research purposes	For research purposes	For research purposes	For research purposes	Occasionally	Occasionally
Hungary	Always	Occasionally	Usually	Usually	Usually	Occasionally	Occasionally	For research purposes	Never	Never	For research purposes	Occasionally	Occasionally
Italy	Always	Always	Always	Always	Usually	Occasionally	Occasionally	For research purposes	For research purposes	For research purposes	Always	Occasionally	Occasionally
Lithuania	Always	Always	Usually	Occasionally	Occasionally	Never	Not available	Not available	Not available	Not available	Occasionally	Never	Never
The Netherlands	Always	Occasionally	Never	Occasionally	Always	Occasionally	Occasionally	Never	For research purposes	For research purposes	Never	For research purposes	Occasionally
Norway	Always	Always	Always	Occasionally	Always	Occasionally	Occasionally	For research purposes	For research purposes	For research purposes	Occasionally	For research purposes	Occasionally
Poland	Usually	Occasionally	Usually	Occasionally	Occasionally	For research purposes	Occasionally	Occasionally	For research purposes	For research purposes	Occasionally	For research purposes	For research purposes
Portugal	Usually	Occasionally	Occasionally	Usually	Occasionally	Occasionally	For research purposes	For research purposes	For research purposes	For research purposes	For research purposes	Occasionally	For research purposes
Sweden	Always	Occasionally	Occasionally	Occasionally	Occasionally	Usually	Occasionally	For research purposes	For research purposes	For research purposes	For research purposes	For research purposes	Occasionally

IHC, immunohistochemistry; large, >50 genes; MSI, microsatellite instability; NGS, next-generation sequencing; PCR, polymerase chain reaction; RNAseq, RNA sequencing; small, <50 genes; TMB, tumor mutational burden; WES, whole-exome sequencing; WGS, whole-genome sequencing.

A uniform rule regarding reimbursement per testing technique was not applicable across countries, as coverage depended on tumor type, patient selection, and national health care systems. In general, 57.1% of the countries (*n* = 8; Denmark, Estonia, Finland, France, Hungary, Norway, Poland, and Sweden) reported that testing techniques were covered by public reimbursement. In Croatia, all testing costs were reimbursed by the patient’s insurance, and in Lithuania and the Netherlands, any remaining costs not reimbursed by the patient’s insurance were covered by the hospital. In Italy, reimbursement depended on the type of testing technique, the region, and clinical setting, while in Germany reimbursement depended on the patient’s insurance status, with costs being funded through either public or private health insurance, or by self-payment of the patient. Funding in Portugal was based on ‘homogeneous diagnostic groups’, whereby predefined amounts were transferred to the hospitals, based on the diagnosis. These amounts were intended to cover all expenses. Due to substantial missing data, results related to pricing of the different testing techniques were inconclusive.

### Patient questionnaire

Between August and December 2024, a total of 288 patients from 16 different countries completed the questionnaire (Supplementary Appendix S3, available at https://doi.org/10.1016/j.esmoop.2025.105872). Baseline patient characteristics are reported in Table 3. Norway accounted for the highest proportion of respondents (*n* = 87, 30.2%), followed by Croatia (*n* = 49, 17.0%). The most frequently reported tumor types were breast cancer (*n* = 105, 36.5%) and ovarian cancer (*n* = 47, 16.3%). Fifty-four percent of patients (*n* = 156) were between 41 and 60 years of age. A total of 209 patients (72.6%) reported higher education as their highest achieved educational level. Most patients were treated in a university hospital or cancer center (*n* = 233, 80.9%) and lived within 30 km of their treating hospital (*n* = 164, 56.9%).

**Table 3:** Baseline characteristics of patient respondents

	Total (*n* = 288)	%
Patient respondents per country		
Norway	87	30.2
Croatia	49	17.0
Poland	31	10.8
Lithuania	23	8.0
Sweden	18	6.3
France	17	5.9
The Netherlands	14	4.9
Finland	12	4.2
Denmark	11	3.8
Italy	9	3.1
Germany	8	2.8
Hungary	3	1.0
Estonia	2	0.7
Spain	2	0.7
Belgium	1	0.3
Portugal	1	0.3
Tumor type		
Breast cancer	105	36.5
Ovarian cancer	47	16.3
Lung cancer	32	11.1
Prostate cancer	22	7.6
Colorectal cancer	21	7.3
Sarcoma	9	3.1
Melanoma	9	3.1
Cervical cancer	7	2.4
Biliary tract cancer	5	1.7
Neuroendocrine tumor	4	1.4
Other^a^	27	9.4
Age, years		
18-25	2	0.7
26-40	19	6.6
41-60	156	54.2
61-75	98	34.0
>75	13	4.5
Education		
Primary education	3	1.0
Secondary education	47	16.3
Vocational education	29	10.1
Short higher education (up to 3 years)	71	24.7
Long higher education (≥4 years)	138	47.9
Treated hospital		
University hospital or cancer center	233	80.9
Regional hospital	49	17.0
Private outpatient clinic	4	1.4
Unknown	2	0.7
Travel distance to hospital (km)		
<10	91	31.6
10-30	73	25.3
30-50	37	12.8
50-100	36	12.5
>100	51	17.7

a Pancreatic cancer, testicular cancer, urothelial cancer, uterine cancer (all *n* = 3), central nervous system cancer, esophageal cancer, gastric cancer, head and neck cancer, lymphoma, small intestine cancer (all *n* = 2), hepatocellular cancer, pleural mesothelioma, thyroid cancer (all *n* = 1).

Of all patients, 252 (87.5%) reported having undergone molecular diagnostics at some point during their cancer trajectory. The remaining patients (*n* = 36, 12.5%) were unsure whether such testing had been carried out. Among those who received molecular diagnostics, it was most frequently carried out at initial diagnosis, before treatment initiation (*n* = 116, 42.5%) and/or in between treatment regimens (*n* = 89, 32.6%). Twenty patients (7.3%) mentioned that testing was carried out after all standard-of-care options had been exhausted. In the vast majority, the treating physician had ordered the tests (*n* = 207, 82.1%), while 24 patients found a facility themselves and requested testing (9.5%).

Of the patients who received molecular diagnostics, two-thirds (*n* = 168, 66.7%) believed the treating physician had explained the potential treatment consequences of molecular diagnostics, while the remaining patients stated that this was not explained (*n* = 68, 27.0%) or were unsure (*n* = 16, 6.3%). Genetic counseling was provided in 30.2% of cases (*n =* 76). Information about the molecular diagnostics procedure was mostly provided by a health care professional (*n* = 180, 62.3%). Some patients reported receiving information through alternative routes, such as through online research (*n* = 48, 16.6%). However, 38 patients (13.1%) reported that they did not receive any information. On a 5-point Likert scale, the median response regarding how well patients felt informed was 3 [moderately informed; interquartile range (IQR) 2-4]. There were no remarkable differences between the countries. Twenty-seven patients (10.7%) reported to have paid, either fully or partially, for the molecular diagnostics themselves and came from nine different countries, namely Belgium, Croatia, Finland, France, Germany, Italy, Lithuania, Norway, and Poland. Twelve patients (4.8%) were unsure how the testing was reimbursed, and for the remaining patients (*n* = 213, 84.5%) the costs were covered either by their insurance, public reimbursement, or the hospital or clinical trial.

Patient responses to knowledge questions on molecular diagnostics are presented in Supplementary Table S5, available at https://doi.org/10.1016/j.esmoop.2025.105872. Over half of the patients (*n* = 160, 55.6%) correctly answered question 1, stating that ‘genes consist of parts of DNA’. Almost two-thirds of patients (*n* = 210, 72.9%) knew that most cancers are not hereditary. However, knowledge on germline testing was lower, since only 90 patients (31.2%) correctly answered that ‘a blood sample or cheek swab’ was used for this. Question 4, regarding the use of molecular diagnostics on tumor material, received at least one of the correct answers 430 times (72.6%). Only one patient (0.3%) answered all questions correctly. When considering at least one of the correct options from question 4, 14 patients (4.9%) provided correct answers to all questions, of whom all but 1 patient (92.9%) reported a higher education level.

Among 34.5% (*n* = 87) of the patients who underwent molecular diagnostics, the turnaround time (TAT) was reportedly between 2 and 4 weeks, while 82 patients (32.5%) experienced a TAT exceeding 4 weeks. Forty patients (15.9%) mentioned a TAT of <2 weeks, including all patients from Denmark. In response to the question if patients would want to be informed if a detected mutation was hereditary, the majority (*n* = 226, 89.7%) answered yes. Twelve patients (4.8%) preferred not to know, and 14 patients (5.6%) were unsure. Ultimately, hereditary mutations were identified in 27 patients (10.7%), while for one-third of patients (*n* = 84, 33.3%) the detected mutations were somatic. The remaining patients were either unsure (*n* = 89, 35.3%) or had no mutations identified (*n* = 52, 20.6%). In the end, a total of 112 patients (44.4%) received targeted treatment based on the molecular testing results, of whom 70 (62.5%) participated in a clinical trial. Twenty-four patients (9.5%) reported a necessary transfer to another hospital for the indicated treatment. Overall satisfaction with the provided information regarding the test results, measured on a 5-point Likert scale, reflected a median score of 3 (reasonably satisfied; IQR 2-4). However, among those who received treatment based on the results, the median satisfaction score was 4 (quite satisfied; IQR 3-5).

The final questions addressed cross-border access to clinical trials. Most patients (*n* = 246, 85.4%) expressed a willingness to travel abroad if necessary to receive appropriate treatment, of whom 38.6% (*n* = 95) indicated that they would do so even in the absence of reimbursement. The most frequently selected suggestion for improving the overall experience with molecular diagnostics was the provision of more understandable information on the topic (*n* = 129, 29.6%), followed by the recommendation to offer genetic counseling before and after testing (*n* = 105, 24.1%).

## Discussion

Since Frederick Sanger developed the first technology for DNA sequencing almost 50 years ago,^14^ enabling the determination of nucleotide sequences, the field of genomics has advanced substantially. These developments have led to an increase in comprehensive testing and, ultimately, targeted treatment options for patients. Despite this progress, unequal access to both genomic testing and targeted therapies persists in the EU. With this study, we aimed to evaluate the current situation of precision oncology from three different perspectives: the medical oncologist, the pathologist, and the patient.

From the perspective of the medical oncologist, access to molecular diagnostics remains unequal in most countries, with only 28.6% reporting equitable access. The most mentioned barriers that withhold countries from carrying out molecular diagnostics include limited access to therapies, reimbursement issues, and the absence of a suitable trial. On the other hand, molecular diagnostics was most often used for patients with metastatic, rare, or unknown cancers, as well as for EMA-approved drugs. This is consistent with previous research,^9^ highlighting the added value of molecular diagnostics in guiding treatment options for more complex cases. Analysis of the different biomarkers tested per tumor type revealed that biomarkers linked to available targeted therapies were tested more frequently. However, tumor-agnostic targets, such as MSI, *NTRK* fusions, or TMB, were less often tested, specifically in tumor types lacking tumor-specific targets, which may reflect the lack of EMA-approved drugs in these alterations. This pattern is in line with the findings from the ESMO study by Bayle et al., which showed that biomarkers with an ESMO Scale of Clinical Actionability for molecular Targets level >2 were mainly tested within research settings.^10^^,^^15^ Limited testing of these biomarkers results in difficulties identifying suitable patients who could potentially benefit from tumor-agnostic treatments. Over the past 5 years, the EMA has approved several of these effective treatments based on specific alterations, regardless of the tumor type, such as larotrectinib and entrectinib for *NTRK* fusions and selpercatinib for *RET* fusions.^16^, ^17^, ^18^

The pathologist questionnaire revealed that 92.3% of the responding countries had implemented an MTB for a multidisciplinary discussion of findings from molecular diagnostics. These findings highlight the rapid evolution of precision oncology in clinical practice, as the first MTB was introduced only a decade ago.^19^ However, 35.7% of the countries reported either a lack of ISO accreditation or very limited (less than half of the laboratories carrying out molecular diagnostics) ISO accreditation, raising concerns about the clinical implications of test results and potential quality risks. In addition, the limited or absent availability of complex sequencing methods in several countries underscored the inequality between countries, as broad testing is often essential to find potential targetable alterations, especially in complex or rare cases. However, as new and expensive targeted therapies keep emerging, health care costs in oncology are expected to keep rising.^20^ To identify eligible patients for these therapies, broad molecular diagnostics is essential. With the growing implementation and infrastructure for complex testing techniques, such as large gene panels and WGS, their associated costs are expected to decrease over time, potentially making it easier for lower-income countries to adopt these techniques as well. Moreover, by implementing broad diagnostics, it is also possible to identify patients who are unlikely to respond to specific therapies upfront, preventing patients from receiving ineffective treatments, while simultaneously reducing health care costs.^21^

From the patient perspective, two-thirds of patients felt that the procedure for molecular diagnostics and its implications for treatment were explained by their health care professional. However, only 30.2% received genetic counseling. Providing this counseling was identified as the second most suggested improvement. The most common recommendation from patients was the desire for more understandable information on the topic. Similar results have been observed by Normanno et al. in 2022, already highlighting the need for providing comprehensible information to patients by educating and training all relevant health care workers.^11^ With only 28.6% of medical oncologists reporting that molecular diagnostics is a mandatory component of the medical oncology specialization in their country, this leaves room for improvement.

Several limitations need to be considered. Firstly, the study design required the formation and use of non-validated questionnaires, allowing for differences in the interpretation of the questions. As we desired one respondent per country for the medical oncologist and pathologist questionnaires, the results rely on the assumption that these respondents accurately reported representative answers from their country. However, differences between or within countries need to be considered. Furthermore, the multiple-choice response options in these questionnaires (never, for research purposes, occasionally, usually, always) were not accompanied by explicit definitions, thereby allowing for subjective interpretation by the respondents. Additionally, as only 15 of the 27 EU member states participated in the PCM4EU consortium, the generalizability of the findings to the entire EU is limited. The number of patient responders varied across countries, including several countries with very few responses, making it difficult to draw country-specific conclusions when interpreting the patient results. The nature of the questionnaire may also have introduced selection bias, as patients willing to answer such an online questionnaire might not be representative of the total population, as well as potential recall and reporting bias. In addition, the clinical impact of molecular diagnostics on treatment outcomes was not evaluated, including how possible delays in testing or disparities in access may influence patient outcomes, and warrants future investigation.

### Conclusions

In conclusion, this study offers a comprehensive snapshot of the implementation of PCM across the EU countries that participated in the PCM4EU consortium, from the point of view of medical oncologists, pathologists, and patients, highlighting persistent inequalities, ranging from disparities in available biomarker testing techniques to their reimbursement, and areas regarding information to patients that need improvement. The PCM4EU consortium addressed these challenges: by facilitating access to similar treatments across countries with the establishment of a DRUP-like clinical trial network, and accelerating data generation by sharing data, patients in Europe are one step closer to equitable access to precision oncology.

An audio PaperClip is available at https://doi.org/10.1016/j.esmoop.2025.105872#mmc1.
