# Supplementary material for: A comprehensive assessment of the existing landscape of personalized cancer medicine in the European Union, on behalf of the PCM4EU consortium
Source: ESMO Open. 2025 Nov 6;10(11):105872. doi: 10.1016/j.esmoop.2025.105872 (PMC12639420; doi:10.1016/j.esmoop.2025.105872)
Supplement: Supplementary Figure 1 [file mmc1.pdf]

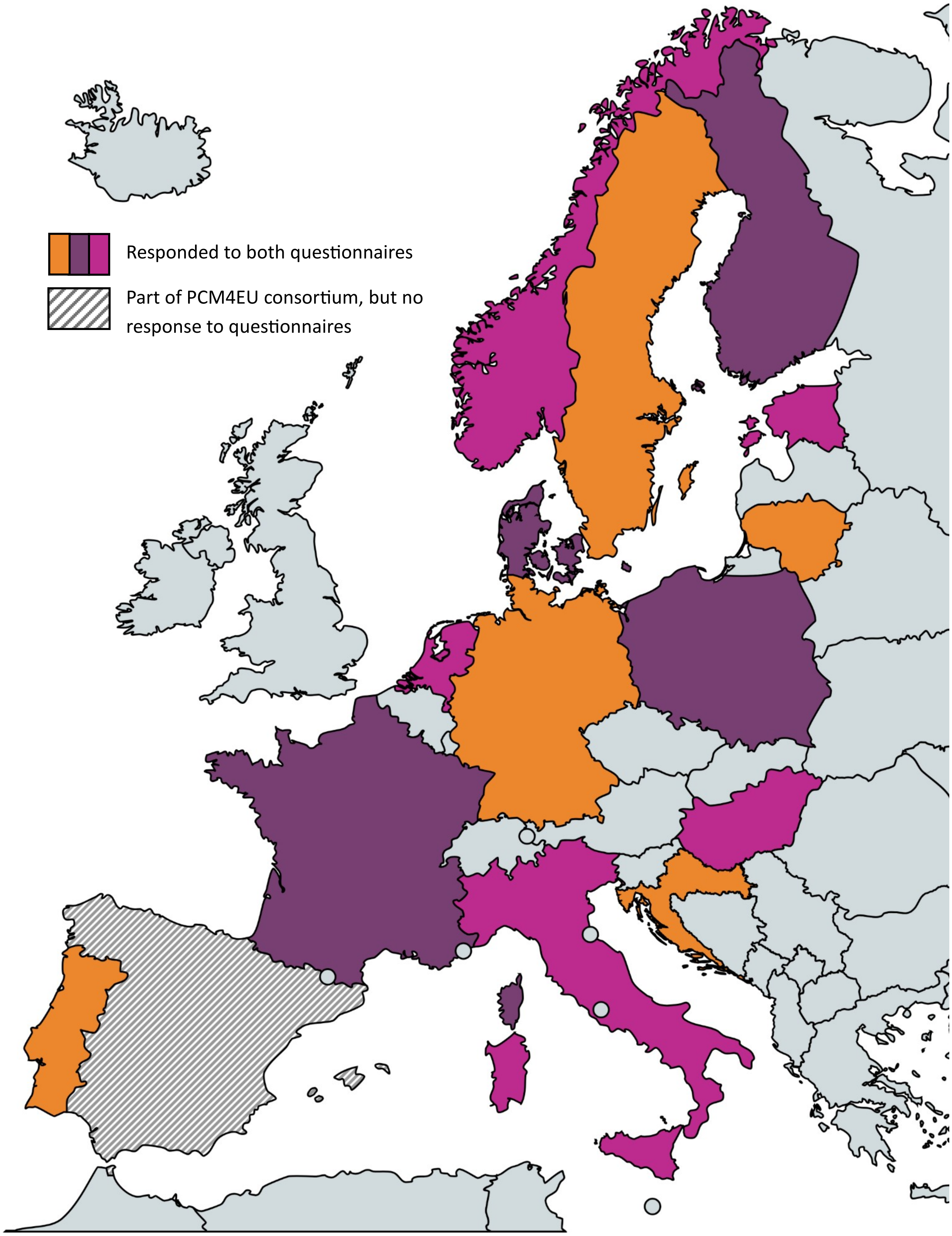

Supplementary Figure S1. Countries within PCM4EU consortium that received the medical oncologist and pathologist questionnaires.

Countries that answered the medical oncologist and pathologist questionnaire were: Croatia, Denmark, Estonia, Finland, France, Germany, Hungary, Italy, Lithuania, the Netherlands, Norway, Poland, Portugal, and Sweden (*n* = 14). Spain did not respond to the questionnaires.
