# Supplementary material for: A comprehensive assessment of the existing landscape of personalized cancer medicine in the European Union, on behalf of the PCM4EU consortium
Source: ESMO Open. 2025 Nov 6;10(11):105872. doi: 10.1016/j.esmoop.2025.105872 (PMC12639420; doi:10.1016/j.esmoop.2025.105872)
Supplement: Supplementary Figure 2 [file mmc2.pdf]

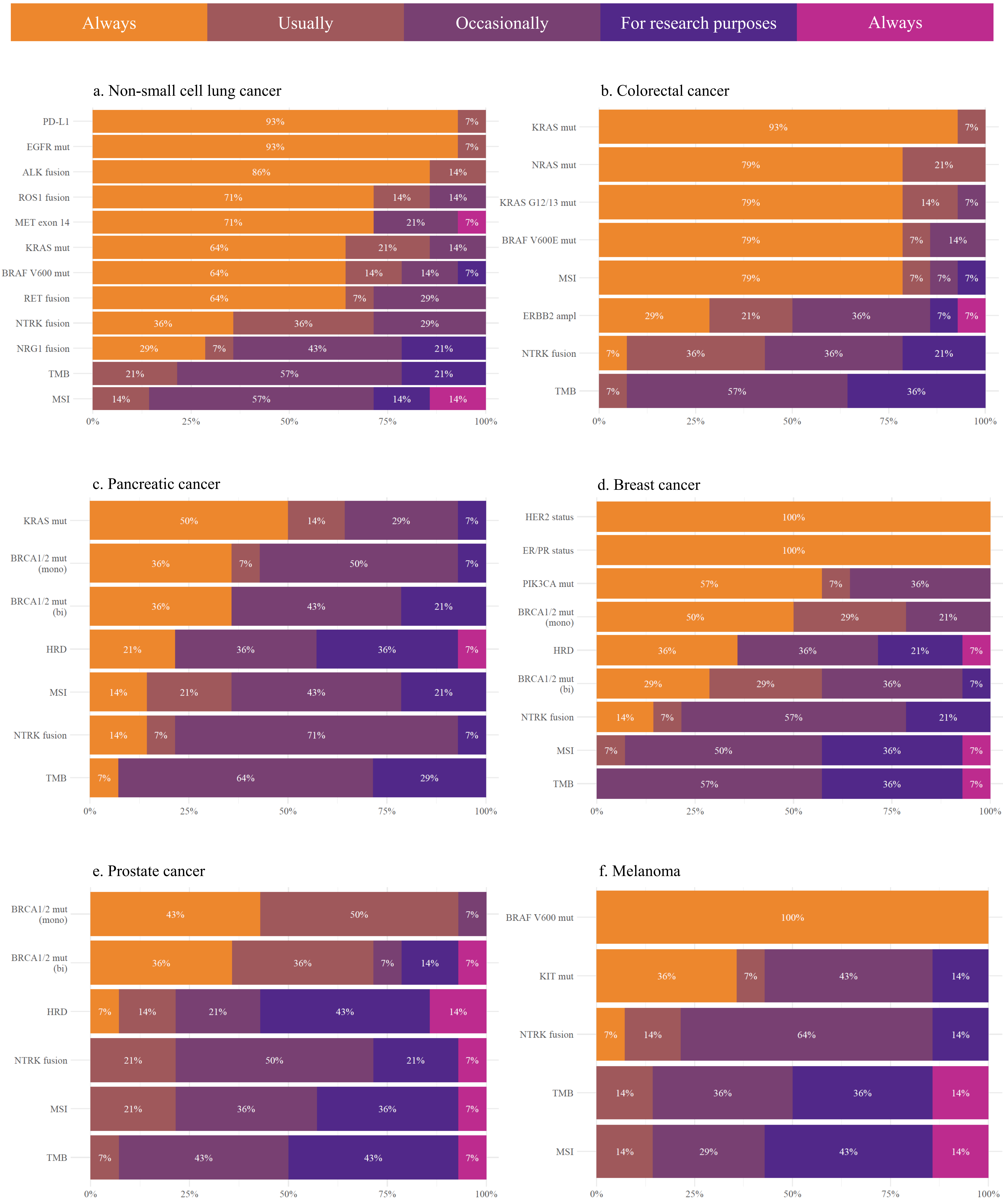

Always
Usually
Occasionally
For research purposes
Always
a. Non-small cell lung cancer
PD-L1 93% 7%
EGFR mut 93% 7%
ALK fusion 86% 14%
ROS1 fusion 71% 14% 14%
MET exon 14 71% 21% 7%
KRAS mut 64% 21% 14%
BRAF V600 mut 64% 14% 14% 7%
RET fusion 64% 7% 29%
NTRK fusion 36% 36% 29%
NRG1 fusion 29% 7% 43% 21%
TMB 21% 57% 21%
MSI 14% 57% 14% 14%
0% 25% 50% 75% 100%
b. Colorectal cancer
KRAS mut 93% 7%
NRAS mut 79% 21%
KRAS G12/13 mut 79% 14% 7%
BRAF V600E mut 79% 7% 14%
MSI 79% 7% 7% 7%
ERBB2 ampl 29% 21% 36% 7% 7%
NTRK fusion 7% 36% 36% 21%
TMB 7% 57% 36%
0% 25% 50% 75% 100%
c. Pancreatic cancer
KRAS mut 50% 14% 29% 7%
BRCA1/2 mut (mono) 36% 7% 50% 7%
BRCA1/2 mut (bi) 36% 43% 21%
HRD 21% 36% 36% 7%
MSI 14% 21% 43% 21%
NTRK fusion 14% 7% 71% 7%
TMB 7% 64% 29%
0% 25% 50% 75% 100%
d. Breast cancer
HER2 status 100%
ER/PR status 100%
PIK3CA mut 57% 7% 36%
BRCA1/2 mut (mono) 50% 29% 21%
HRD 36% 36% 21% 7%
BRCA1/2 mut (bi) 29% 29% 36% 7%
NTRK fusion 14% 7% 57% 21%
MSI 7% 50% 36% 7%
TMB 57% 36% 7%
0% 25% 50% 75% 100%
e. Prostate cancer
BRCA1/2 mut (mono) 43% 50% 7%
BRCA1/2 mut (bi) 36% 36% 7% 14% 7%
HRD 7% 14% 21% 43% 14%
NTRK fusion 21% 50% 21% 7%
MSI 21% 36% 36% 7%
TMB 7% 43% 43% 7%
0% 25% 50% 75% 100%
f. Melanoma
BRAF V600 mut 100%
KIT mut 36% 7% 43% 14%
NTRK fusion 7% 14% 64% 14%
TMB 14% 36% 36% 14%
MSI 14% 29% 43% 14%
0% 25% 50% 75% 100%

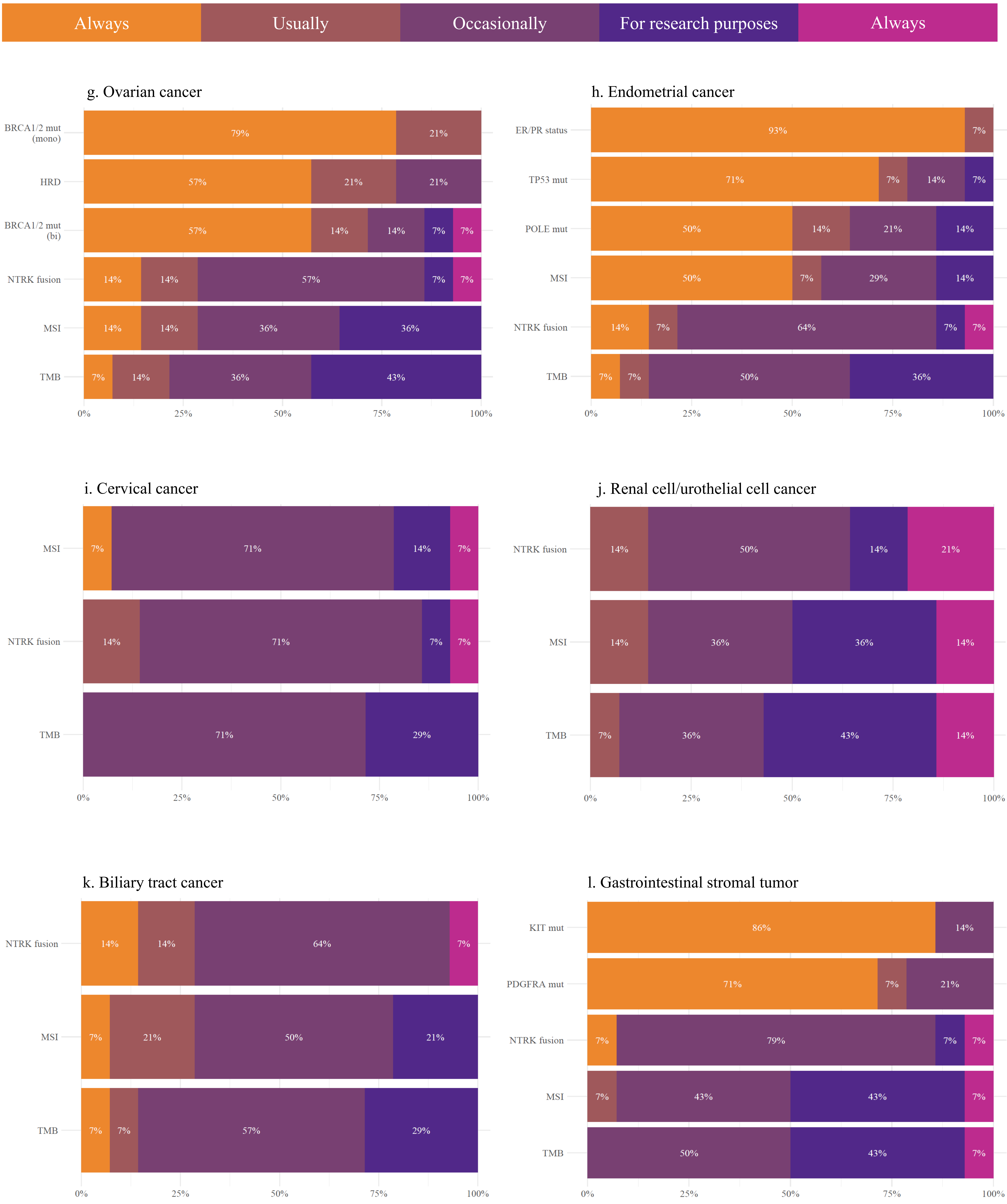
Always
Usually
Occasionally
For research purposes
Always
g. Ovarian cancer
BRCA1/2 mut (mono)
79%
21%
HRD
57%
21%
21%
BRCA1/2 mut (bi)
57%
14%
14%
7%
7%
NTRK fusion
14%
14%
57%
7%
7%
MSI
14%
14%
36%
36%
TMB
7%
14%
36%
43%
0%
25%
50%
75%
100%
h. Endometrial cancer
ER/PR status
93%
7%
TP53 mut
71%
7%
14%
7%
POLE mut
50%
14%
21%
14%
MSI
50%
7%
29%
14%
NTRK fusion
14%
7%
64%
7%
7%
TMB
7%
7%
50%
36%
0%
25%
50%
75%
100%
i. Cervical cancer
MSI
7%
71%
14%
7%
NTRK fusion
14%
71%
7%
7%
TMB
71%
29%
0%
25%
50%
75%
100%
j. Renal cell/urothelial cell cancer
NTRK fusion
14%
50%
14%
21%
MSI
14%
36%
36%
14%
TMB
7%
36%
43%
14%
0%
25%
50%
75%
100%
k. Biliary tract cancer
NTRK fusion
14%
14%
64%
7%
MSI
7%
21%
50%
21%
TMB
7%
7%
57%
29%
0%
25%
50%
75%
100%
l. Gastrointestinal stromal tumor
KIT mut
86%
14%
PDGFRA mut
71%
7%
21%
NTRK fusion
7%
79%
7%
7%
MSI
7%
43%
43%
7%
TMB
50%
43%
7%
0%
25%
50%
75%
100%

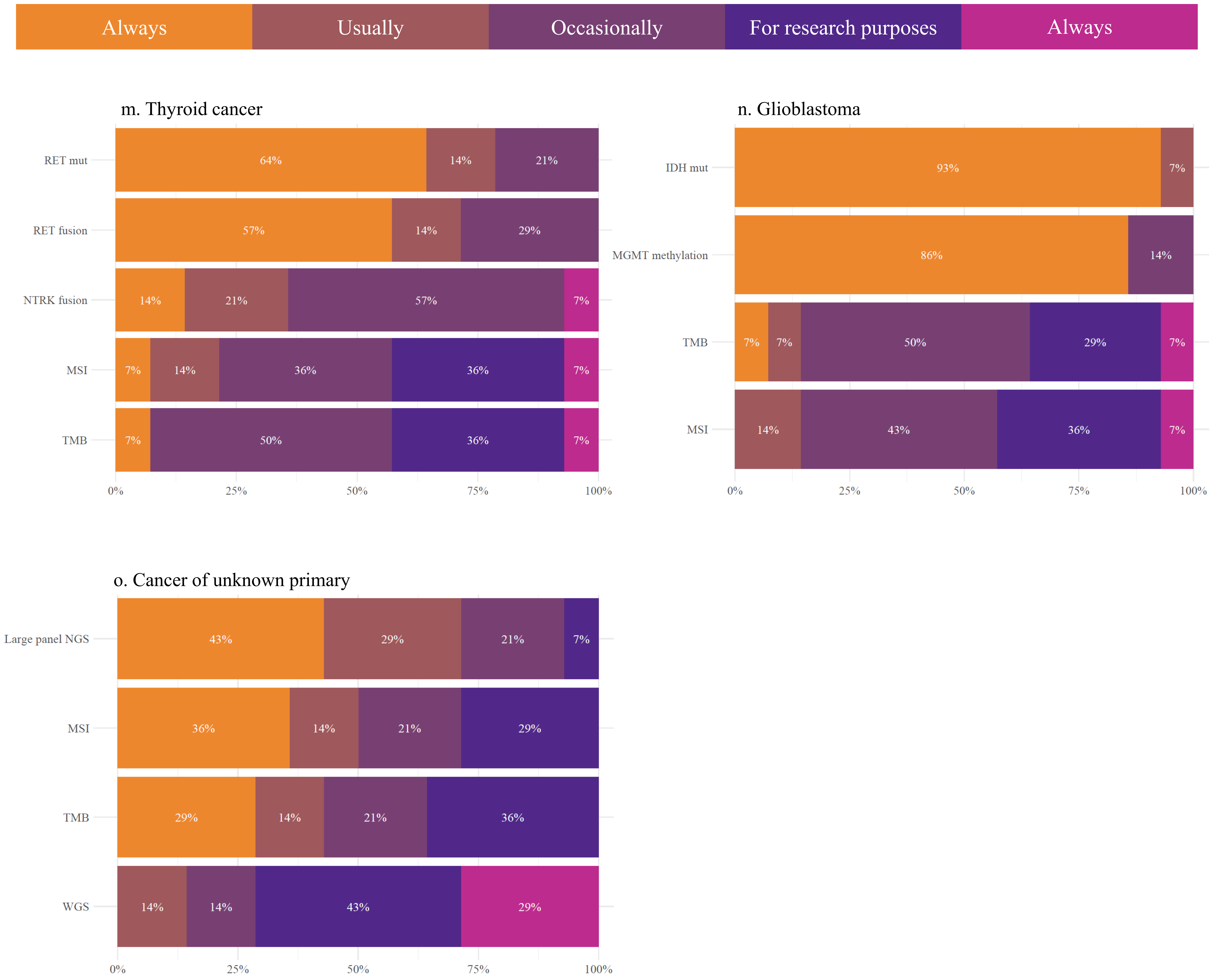

Supplementary Figure S2. Biomarker usage across tumor types.
Bar plots showing biomarker usage across tumor types with grouped answers from all countries. Abbreviations: mut, mutation; TMB, tumor mutational burden; MSI, microsatellite instability; ampl, amplification; mono, mono-allelic; bi, bi-allelic; HRD, homologous recombination deficiency; NGS, next-generation sequencing; WGS, whole-genome sequencing.
