# Supplementary material for: A comprehensive assessment of the existing landscape of personalized cancer medicine in the European Union, on behalf of the PCM4EU consortium
Source: ESMO Open. 2025 Nov 6;10(11):105872. doi: 10.1016/j.esmoop.2025.105872 (PMC12639420; doi:10.1016/j.esmoop.2025.105872)
Supplement: Supplementary Figure 3 [file mmc3.pdf]

Supplementary Figure S3. Types of laboratories performing biomarker testing techniques.

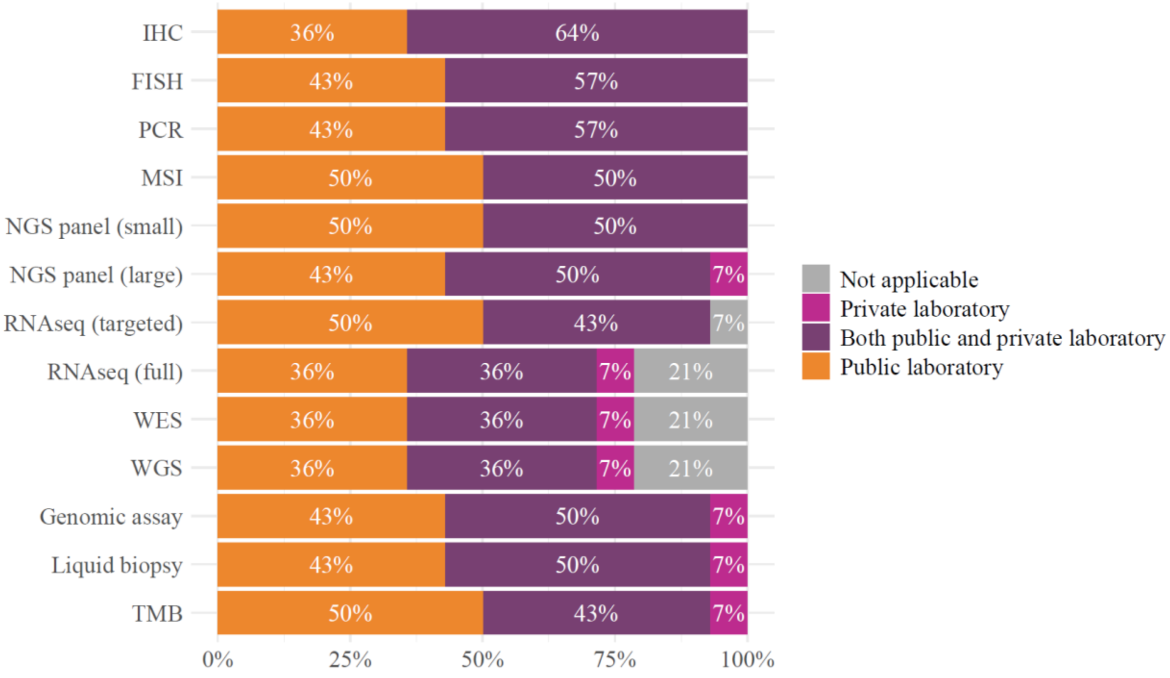

Figure legend. Bar plot showing grouped answers of all countries regarding types of laboratories performing different biomarker testing techniques. Abbreviations: IHC, immunohistochemistry; FISH, fluorescence in situ hybridization; PCR, polymerase chain reaction; MSI, microsatellite instability; NGS, next-generation sequencing; small, <50 genes; large, >50 genes; RNAseq, RNA sequencing; WES, whole-exome sequencing; WGS, whole-genome sequencing; TMB, tumor mutational burden.
