# Supplementary material for: A comprehensive assessment of the existing landscape of personalized cancer medicine in the European Union, on behalf of the PCM4EU consortium
Source: ESMO Open. 2025 Nov 6;10(11):105872. doi: 10.1016/j.esmoop.2025.105872 (PMC12639420; doi:10.1016/j.esmoop.2025.105872)
Supplement: Supplementary Material [file mmc4.docx]

**Supplementary Material**

Table of Contents

[Supplementary Table S1. When and for what reason molecular diagnostics are performed. 2](#_Toc212478523)

[Supplementary Table S2. Barriers for performing molecular diagnostics. 3](#_Toc212478524)

[Supplementary Table S3. Barriers for reimbursement of molecular diagnostics. 4](#_Toc212478525)

[Supplementary Table S4. Types of laboratories performing biomarker testing techniques. 5](#_Toc212478526)

[Supplementary Table S5. Patient responses to knowledge questions on molecular diagnostics (n = 288). 6](#_Toc212478527)

[Supplementary Appendix S1. Medical oncologist questionnaire 7](#_Toc212478528)

[Supplementary Appendix S2. Pathologist questionnaire 16](#_Toc212478529)

[Supplementary Appendix S3. Patient questionnaire 27](#_Toc212478530)

# Supplementary Table S1. When and for what reason molecular diagnostics are performed.

| **Country** | **At initial diagnosis for all patients** | **At initial diagnosis for high risk patients** | **At initial diagnosis for metastatic patients** | **To investigate treatment options in patients who exhausted all standard of care options** | **To investigate treatment options in rare malignancies** | **If the malignancy was of unknown origin** | **For the use of EMA-approved drugs** | **For off-label use** | **To provide diagnostic information** | **To provide prognostic information** | **For eligibility for clinical trials** |
| --- | --- | --- | --- | --- | --- | --- | --- | --- | --- | --- | --- |
| Croatia | Occasionally | Occasionally | Usually | Usually | Usually | Usually | Usually | Usually | Usually | Usually | Usually |
| Denmark | Occasionally | Usually | Usually | Usually | Usually | Occasionally | Usually | For research purposes | Occasionally | Occasionally | For research purposes |
| Estonia | Occasionally | Always | Occasionally | Usually | Usually | Always | Always | Occasionally | Occasionally | Occasionally | Occasionally |
| Finland | Occasionally | Occasionally | Occasionally | For research purposes | Usually | Usually | Usually | For research purposes | For research purposes | For research purposes | For research purposes |
| France | For research purposes | Occasionally | Usually | Usually | Usually | Usually | Always | For research purposes | Occasionally | For research purposes | Usually |
| Germany | Never | Never | Usually | Usually | Occasionally | Usually | Always | Usually | Usually | Occasionally | Usually |
| Hungary | Occasionally | Usually | Always | Always | Always | Always | Usually | Occasionally | Occasionally | Usually | Occasionally |
| Italy | Occasionally | Occasionally | Usually | Always | Always | Usually | Usually | Always | Occasionally | For research purposes | Always |
| Lithuania | Occasionally | Occasionally | Occasionally | Usually | Usually | Occasionally | Usually | Occasionally | Occasionally | Occasionally | Occasionally |
| Netherlands | Occasionally | Usually | Usually | Usually | Usually | Always | Always | Usually | Usually | Occasionally | Usually |
| Norway | Never | Never | Occasionally | Occasionally | Occasionally | Occasionally | Always | Never | Never | Never | Occasionally |
| Poland | Occasionally | Usually | Usually | Usually | Occasionally | Usually | Occasionally | Occasionally | Usually | Occasionally | Occasionally |
| Portugal | Never | Never | Occasionally | Usually | Usually | Usually | Always | Occasionally | Occasionally | Occasionally | Occasionally |
| Sweden | Occasionally | Usually | Usually | Usually | Usually | Usually | Usually | Occasionally | Occasionally | Occasionally | Usually |

| **Country** | **The molecular testing procedure is not reimbursed** | **The drug is not reimbursed** | **There is no suitable drug available (e.g. off-label)** | **Difficulty to find a suitable clinical trial** | **Lack of experience with the procedure** | **Lack of experience with the interpretation of test results** | **Lack of evidence for effectiveness of the test** | **Long turnaround time for test results** | **Ethical considerations (e.g. incidental results** |
| --- | --- | --- | --- | --- | --- | --- | --- | --- | --- |
| Croatia | Occasionally | Occasionally | Rarely | Occasionally | Rarely | Occasionally | Occasionally | Occasionally | Occasionally |
| Denmark | Rarely | Frequently | Frequently | Occasionally | Frequently | Frequently | Frequently | Frequently | Rarely |
| Estonia | Never | Never | Never | Never | Never | Never | Never | Never | Never |
| Finland | Always | Occasionally | Occasionally | Occasionally | Occasionally | Occasionally | Occasionally | Rarely | Rarely |
| France | Rarely | Occasionally | Occasionally | Frequently | Rarely | Rarely | Frequently | Occasionally | Rarely |
| Germany | Frequently | Occasionally | Occasionally | Occasionally | Rarely | Occasionally | Occasionally | Occasionally | Rarely |
| Hungary | Rarely | Frequently | Frequently | Frequently | Occasionally | Rarely | Rarely | Occasionally | Rarely |
| Italy | Occasionally | Frequently | Frequently | Occasionally | Rarely | Never | Rarely | Occasionally | Occasionally |
| Lithuania | Frequently | Frequently | Frequently | Frequently | Occasionally | Rarely | Rarely | Occasionally | Occasionally |
| Netherlands | Rarely | Rarely | Rarely | Rarely | Rarely | Rarely | Rarely | Rarely | Rarely |
| Norway | Frequently | Occasionally | Frequently | Frequently | Occasionally | Rarely | Frequently | Occasionally | Never |
| Poland | Occasionally | Frequently | Occasionally | Frequently | Occasionally | Occasionally | Occasionally | Frequently | Rarely |
| Portugal | Rarely | Occasionally | Occasionally | Rarely | Rarely | Rarely | Rarely | Occasionally | Rarely |
| Sweden | Occasionally | Occasionally | Occasionally | Frequently | Rarely | Never | Occasionally | Rarely | Rarely |

# Supplementary Table S2. Barriers for performing molecular diagnostics.

| **Country** | **The drug that targets the specific biomarker is not available** | **The drug that targets the specific biomarker is not reimbursed** | **Lack of evidence that the tests have enough medical implication** | **The specific mutation is too rare** |
| --- | --- | --- | --- | --- |
| Croatia | Frequently | Frequently | Rarely | Occasionally |
| Denmark | Frequently | Frequently | Frequently | Frequently |
| Estonia | Never | Never | Never | Never |
| Finland | Occasionally | Occasionally | Frequently | Frequently |
| France | Occasionally | Occasionally | Frequently | Occasionally |
| Germany | Rarely | Frequently | Frequently | Rarely |
| Hungary | Frequently | Frequently | Occasionally | Occasionally |
| Italy | Occasionally | Frequently | Occasionally | Rarely |
| Lithuania | Rarely | Rarely | Never | Occasionally |
| Netherlands | Occasionally | Occasionally | Rarely | Rarely |
| Norway | Occasionally | Occasionally | Frequently | Occasionally |
| Poland | Occasionally | Frequently | Occasionally | Frequently |
| Portugal | Occasionally | Occasionally | Rarely | Occasionally |
| Sweden | Occasionally | Occasionally | Occasionally | Occasionally |

# Supplementary Table S3. Barriers for reimbursement of molecular diagnostics.

# Supplementary Table S4. Types of laboratories performing biomarker testing techniques.

| **Country** | **IHC** | **FISH** | **PCR** | **MSI** | **NGS panel (small)** | **NGS panel**  **(large)** | **RNAseq**  **(targeted)** | **RNAseq**  **(full)** | **WES** | **WGS** | **Genomic assay** | **Liquid biopsy** | **TMB** |
| --- | --- | --- | --- | --- | --- | --- | --- | --- | --- | --- | --- | --- | --- |
| Croatia | Public | Public | Public | Public | Public | Public | Public | N/A | N/A | N/A | N/A | Public | Public |
| Denmark | Public | Public | Public | Public | Public | Public | Public | Public | Public | Public | Public | Public | Public |
| Estonia | Public | Public | Both | Public | Public | Public | Public | N/A | N/A | N/A | Public | Private | Public |
| Finland | Both public and private | Both public and private | Public | Public | Public | Both public and private | Public | Public | Public | Public | Both public and private | Both public and private | Both public and private |
| France | Both public and private | Both public and private | Both public and private | Both public and private | Both public and private | Both public and private | Both public and private | Both public and private | Both public and private | Both public and private | Both public and private | Both public and private | Both public and private |
| Germany | Both public and private | Both public and private | Both public and private | Both public and private | Both public and private | Both public and private | Both public and private | Both public and private | Both public and private | Both public and private | Both public and private | Both public and private | Both public and private |
| Hungary | Both public and private | Both public and private | Both public and private | Both public and private | Both public and private | Both public and private | Both public and private | Private | Private | Private | Private | Both public and private | Both public and private |
| Italy | Both public and private | Both public and private | Both public and private | Both public and private | Both public and private | Both public and private | Both public and private | Both public and private | Both public and private | Both public and private | Both public and private | Both public and private | Both public and private |
| Lithuania | Both public and private | Both public and private | Both public and private | Both public and private | Both public and private | Private | N/A | N/A | N/A | N/A | Both public and private | Both public and private | Private |
| Netherlands | Public | Public | Public | Public | Public | Public | Public | Public | Public | Public | Public | Public | Public |
| Norway | Both public and private | Public | Public | Public | Public | Public | Public | Public | Public | Public | Public | Public | Public |
| Poland | Both public and private | Both public and private | Both public and private | Both public and private | Both public and private | Both public and private | Both public and private | Both public and private | Both public and private | Both public and private | Both public and private | Both public and private | Both public and private |
| Portugal | Public | Public | Public | Public | Public | Public | Public | Both public and private | Both public and private | Both public and private | Both public and private | Public | Public |
| Sweden | Both public and private | Both public and private | Both public and private | Both public and private | Both public and private | Both public and private | Both public and private | Public | Public | Public | Public | Public | Public |

**Table legend:** abbreviations: IHC, immunohistochemistry; FISH, fluorescence in situ hybridization; PCR, polymerase chain reaction; MSI, microsatellite instability; NGS, next-generation sequencing; small, <50 genes; large, >50 genes; RNAseq, RNA sequencing; WES, whole-exome sequencing; WGS, whole-genome sequencing; TMB, tumor mutational burden; N/A, not applicable.

# Supplementary Table S5. Patient responses to knowledge questions on molecular diagnostics (n = 288).

| Questions | Correct  *n* (%) | Incorrect  *n* (%) | Unsure  *n* (%) |
| --- | --- | --- | --- |
| 1. Which of the following statements is true? |  |  | 73 (25.3) |
| - Genes consist of parts of DNA | 160 (55.6) |  |  |
| - Cancer is always aggressive |  | 7 (2.4) |  |
| - If a mutation happens in a cell, this will  always result in the development of cancer |  | 32 (11.1) |  |
| - All of the above |  | 16 (5.6) |  |
| 2. The majority of cancer is hereditary |  |  | 57 (19.8) |
| True |  | 21 (7.3) |  |
| False | 210 (72.9) |  |  |
| 3. Testing for germline mutations happens via: |  |  | 72 (25.0) |
| - A blood sample or cheek swab | 90 (31.2) |  |  |
| - Tumor sample |  | 31 (10.8) |  |
| - It can be tested in both ways |  | 95 (33.0) |  |
| 4. Molecular testing of a tumor can be used for:  *(more answers possible*)* |  |  | 25 (4.2) |
| - Identification of a cancer type | 178 (30.1) |  |  |
| - Prediction of someone’s risk to develop  cancer |  | 137 (23.1) |  |
| - Prediction if someone will respond to a  particular treatment | 188 (31.8) |  |  |
| - Prediction of disease outcome | 64 (10.8) |  |  |

**Table legend**: *Total number of responses exceeds the number of patients, as multiple responses per patient were allowed. Abbreviations: DNA, deoxyribonucleic acid.

#
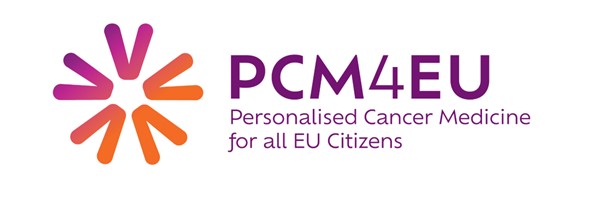
Supplementary Appendix S1. Medical oncologist questionnaire

*Introduction*

Thank you for taking the time to fill in this questionnaire. The questionnaire is intended for medical oncologists who are affiliated with a DRUP-like clinical trial in their country or want to start a DRUP-like clinical trial, all contributing in the PCM4EU project, and consists of questions regarding the use and reimbursement of molecular testing.

The questionnaire is divided into four topics. The first part contains two general questions, the second part includes questions on the availability, use and reimbursement of specific biomarkers per tumour type (15 in total), based on the Dutch List of Minimally Clinically Necessary Molecular Testing. Questions on possible barriers are enclosed in part three and the fourth part consists of questions regarding the use of molecular testing.

It will take around 30 minutes to complete the questionnaire. It is possible to close the questionnaire and finish it at a later stage. The answers will be saved.

By checking the box below you agree that your results will be saved and used for research purposes.

- I agree

I. GENERAL

1. Which country do you live in?
   - Croatia
   - Denmark
   - Estonia
   - Finland
   - France
   - Germany
   - Hungary
   - Italy
   - Lithuania
   - Norway
   - Poland
   - Portugal
   - Spain
   - Sweden
   - The Netherlands
   - Other, namely …………………….
2. Is molecular testing part of the education to become a medical specialist in your country?
   - Yes, it is part of the mandatory curriculum
   - Yes, there are lectures, seminars and symposia on the topic, but this is not mandatory
   - Yes, there is a separate course on the topic, but this is not mandatory
   - No
   - I do not know
   - Other, namely ………………………

II. BIOMARKERS AND REIMBURSEMENT

The following questions address the availability and use of biomarkers per tumour type and if they are reimbursed. Please choose the most suiting option. If there are differences within regions in your country, you can choose multiple options. After each tumour type there is room for additional remarks to specify the regions and elaborate on the options.

1. How would you score the availability and use of the following biomarkers tested for locally advanced or metastatic **non-small cell lung cancer (NSCLC)** in your country and are they reimbursed?

| **Biomarkers for NSCLC** | **Never** | **Research** | **Occasionally** | **Usually** | **Always** | **Reimbursed?** |
| --- | --- | --- | --- | --- | --- | --- |
| - PD-L1 |  |  |  |  |  | Yes / No |
| - EGFR mutations |  |  |  |  |  | Yes / No |
| - BRAF V600 mutations |  |  |  |  |  | Yes / No |
| - KRAS mutations |  |  |  |  |  | Yes / No |
| - MET exon 14 skipping |  |  |  |  |  | Yes / No |
| - ROS1 fusion |  |  |  |  |  | Yes / No |
| - NRG1 fusion |  |  |  |  |  | Yes / No |
| - RET fusion |  |  |  |  |  | Yes / No |
| - ALK fusion |  |  |  |  |  | Yes / No |
| - NTRK fusion |  |  |  |  |  | Yes / No |
| - Tumour Mutational Burden |  |  |  |  |  | Yes / No |
| - Microsatellite instability |  |  |  |  |  | Yes / No |
| - Other, namely… |  |  |  |  |  | Yes / No |

Comments:

…………………………………………………………………………………………………………………………………………………………………………………………………………………………………………………………………………………………………………………………………………………………

1. How would you score the availability and use of the following biomarkers tested for locally advanced or metastatic **colorectal cancer (CRC)** in your country and are they reimbursed?

| **Biomarkers for CRC** | **Never** | **Research** | **Occasionally** | **Usually** | **Always** | **Reimbursed?** |
| --- | --- | --- | --- | --- | --- | --- |
| - KRAS mutations |  |  |  |  |  | Yes / No |
| - KRAS G12/13 mutations |  |  |  |  |  | Yes / No |
| - NRAS mutations |  |  |  |  |  | Yes / No |
| - ERBB2 amplification |  |  |  |  |  | Yes / No |
| - BRAF V600E mutations |  |  |  |  |  | Yes / No |
| - NTRK fusion |  |  |  |  |  | Yes / No |
| - Tumour Mutational Burden |  |  |  |  |  | Yes / No |
| - Microsatellite instability |  |  |  |  |  | Yes / No |
| - Other, namely… |  |  |  |  |  | Yes / No |

Comments:

………………………………………………………………………………………………………………………………………………..…………………………………………………………………………………………….…………………………………………………..……………………………………………………

1. How would you score the availability and use of the following biomarkers tested for locally advanced or metastatic **pancreatic cancer** in your country and are they reimbursed?

| **Biomarkers for pancreatic cancer** | **Never** | **Research** | **Occasionally** | **Usually** | **Always** | **Reimbursed?** |
| --- | --- | --- | --- | --- | --- | --- |
| - KRAS mutations |  |  |  |  |  | Yes / No |
| - Monoallelic BRCA 1/2 mutations |  |  |  |  |  | Yes / No |
| - Biallelic BRCA 1/2 mutations |  |  |  |  |  | Yes / No |
| - Homologous Recombination Deficiency |  |  |  |  |  | Yes / No |
| - NTRK fusion |  |  |  |  |  | Yes / No |
| - Tumour Mutational Burden |  |  |  |  |  | Yes / No |
| - Microsatellite instability |  |  |  |  |  | Yes / No |
| - Other, namely… |  |  |  |  |  | Yes / No |

Comments:

………………………………………………………………………………………………………………………………………………..………………………………………………………………………………………………………………………………………………..……………………………………………………

1. How would you score the availability and use of the following biomarkers tested for locally advanced or metastatic **breast cancer** in your country and are they reimbursed?

| **Biomarkers for breast cancer** | **Never** | **Research** | **Occasionally** | **Usually** | **Always** | **Reimbursed?** |
| --- | --- | --- | --- | --- | --- | --- |
| - ER/PR status |  |  |  |  |  | Yes / No |
| - HER2 status |  |  |  |  |  | Yes / No |
| - Monoallelic BRCA 1/2 mutations |  |  |  |  |  | Yes / No |
| - Biallelic BRCA 1/2 mutations |  |  |  |  |  | Yes / No |
| - PIK3CA mutations |  |  |  |  |  | Yes / No |
| - Homologous Recombination Deficiency |  |  |  |  |  | Yes / No |
| - NTRK fusion |  |  |  |  |  | Yes / No |
| - Tumour Mutational Burden |  |  |  |  |  | Yes / No |
| - Microsatellite instability |  |  |  |  |  | Yes / No |
| - Other, namely… |  |  |  |  |  | Yes / No |

Comments:

………………………………………………………………………………………………………………………………………………..………………………………………………………………………………………………………………………………………………..……………………………………………………

1. How would you score the availability and use of the following biomarkers tested for locally advanced or metastatic **prostate cancer** in your country and are they reimbursed?

| **Biomarkers for prostate cancer** | **Never** | **Research** | **Occasionally** | **Usually** | **Always** | **Reimbursed?** |
| --- | --- | --- | --- | --- | --- | --- |
| - Monoallelic BRCA 1/2 mutations |  |  |  |  |  | Yes / No |
| - Biallelic BRCA 1/2 mutations |  |  |  |  |  | Yes / No |
| - Homologous Recombination Deficiency |  |  |  |  |  | Yes / No |
| - NTRK fusion |  |  |  |  |  | Yes / No |
| - Tumour Mutational Burden |  |  |  |  |  | Yes / No |
| - Microsatellite instability |  |  |  |  |  | Yes / No |
| - Other, namely… |  |  |  |  |  | Yes / No |

Comments:

………………………………………………………………………………………………………………………………………………..………………………………………………………………………………………………………………………………………………..……………………………………………………

1. How would you score the availability and use of the following biomarkers tested for locally advanced or metastatic **melanoma** in your country and are they reimbursed?

| **Biomarkers for melanoma** | **Never** | **Research** | **Occasionally** | **Usually** | **Always** | **Reimbursed?** |
| --- | --- | --- | --- | --- | --- | --- |
| - BRAF V600 mutations |  |  |  |  |  | Yes / No |
| - KIT mutations |  |  |  |  |  | Yes / No |
| - NTRK fusion |  |  |  |  |  | Yes / No |
| - Tumour Mutational Burden |  |  |  |  |  | Yes / No |
| - Microsatellite instability |  |  |  |  |  | Yes / No |
| - Other, namely… |  |  |  |  |  | Yes / No |

Comments:

………………………………………………………………………………………………………………………………………………..………………………………………………………………………………………………………………………………………………..……………………………………………………

1. How would you score the availability and use of the following biomarkers tested for locally advanced or metastatic **ovarian cancer** in your country and are they reimbursed?

| **Biomarkers for ovarian cancer** | **Never** | **Research** | **Occasionally** | **Usually** | **Always** | **Reimbursed?** |
| --- | --- | --- | --- | --- | --- | --- |
| - Monoallelic BRCA 1/2 mutations |  |  |  |  |  | Yes / No |
| - Biallelic BRCA 1/2 mutations |  |  |  |  |  | Yes / No |
| - Homologous Recombination Deficiency |  |  |  |  |  | Yes / No |
| - NTRK fusion |  |  |  |  |  | Yes / No |
| - Tumour Mutational Burden |  |  |  |  |  | Yes / No |
| - Microsatellite instability |  |  |  |  |  | Yes / No |
| - Other, namely… |  |  |  |  |  | Yes / No |

Comments:

………………………………………………………………………………………………………………………………………………..………………………………………………………………………………………………………………………………………………..……………………………………………………

1. How would you score the availability and use of the following biomarkers tested for locally advanced or metastatic **endometrial cancer** in your country and are they reimbursed?

| **Biomarkers for endometrial cancer** | **Never** | **Research** | **Occasionally** | **Usually** | **Always** | **Reimbursed?** |
| --- | --- | --- | --- | --- | --- | --- |
| - ER/PR status |  |  |  |  |  | Yes / No |
| - POLE mutations |  |  |  |  |  | Yes / No |
| - TP53 mutations |  |  |  |  |  | Yes / No |
| - NTRK fusion |  |  |  |  |  | Yes / No |
| - Tumour Mutational Burden |  |  |  |  |  | Yes / No |
| - Microsatellite instability |  |  |  |  |  | Yes / No |
| - Other, namely… |  |  |  |  |  | Yes / No |

Comments:

………………………………………………………………………………………………………………………………………………..………………………………………………………………………………………………………………………………………………..……………………………………………………

1. How would you score the availability and use of the following biomarkers tested for locally advanced or metastatic **cervical cancer** in your country and are they reimbursed?

| **Biomarkers for cervical cancer** | **Never** | **Research** | **Occasionally** | **Usually** | **Always** | **Reimbursed?** |
| --- | --- | --- | --- | --- | --- | --- |
| - NTRK fusion |  |  |  |  |  | Yes / No |
| - Tumour Mutational Burden |  |  |  |  |  | Yes / No |
| - Microsatellite instability |  |  |  |  |  | Yes / No |
| - Other, namely… |  |  |  |  |  | Yes / No |

Comments:

………………………………………………………………………………………………………………………………………………..………………………………………………………………………………………………………………………………………………..……………………………………………………

1. How would you score the availability and use of the following biomarkers tested for locally advanced or metastatic **renal cell/urothelial cell cancer** in your country and are they reimbursed?

| **Biomarkers for renal cell/urothelial cell cancer** | **Never** | **Research** | **Occasionally** | **Usually** | **Always** | **Reimbursed?** |
| --- | --- | --- | --- | --- | --- | --- |
| - NTRK fusion |  |  |  |  |  | Yes / No |
| - Tumour Mutational Burden |  |  |  |  |  | Yes / No |
| - Microsatellite instability |  |  |  |  |  | Yes / No |
| - Other, namely… |  |  |  |  |  | Yes / No |

Comments:

………………………………………………………………………………………………………………………………………………..………………………………………………………………………………………………………………………………………………..……………………………………………………

1. How would you score the availability and use of the following biomarkers tested for locally advanced or metastatic **biliary tract cancer** in your country and are they reimbursed?

| **Biomarkers for biliary tract cancer** | **Never** | **Research** | **Occasionally** | **Usually** | **Always** | **Reimbursed?** |
| --- | --- | --- | --- | --- | --- | --- |
| - NTRK fusion |  |  |  |  |  | Yes / No |
| - Tumour Mutational Burden |  |  |  |  |  | Yes / No |
| - Microsatellite instability |  |  |  |  |  | Yes / No |
| - Other, namely… |  |  |  |  |  | Yes / No |

Comments:

………………………………………………………………………………………………………………………………………………..………………………………………………………………………………………………………………………………………………..……………………………………………………

1. How would you score the availability and use of the following biomarkers tested for locally advanced or metastatic **gastrointestinal stromal tumour (GIST**) in your country and are they reimbursed?

| **Biomarkers for GIST** | **Never** | **Research** | **Occasionally** | **Usually** | **Always** | **Reimbursed?** |
| --- | --- | --- | --- | --- | --- | --- |
| - KIT mutations |  |  |  |  |  | Yes / No |
| - PDGFRα mutations |  |  |  |  |  | Yes / No |
| - NTRK fusion |  |  |  |  |  | Yes / No |
| - Tumour Mutational Burden |  |  |  |  |  | Yes / No |
| - Microsatellite instability |  |  |  |  |  | Yes / No |
| - Other, namely… |  |  |  |  |  | Yes / No |

Comments:

………………………………………………………………………………………………………………………………………………..………………………………………………………………………………………………………………………………………………..……………………………………………………

1. How would you score the availability and use of the following biomarkers tested for locally advanced or metastatic **thyroid cancer** in your country and are they reimbursed?

| **Biomarkers for thyroid cancer** | **Never** | **Research** | **Occasionally** | **Usually** | **Always** | **Reimbursed?** |
| --- | --- | --- | --- | --- | --- | --- |
| - RET mutations |  |  |  |  |  | Yes / No |
| - RET fusions |  |  |  |  |  | Yes / No |
| - NTRK fusion |  |  |  |  |  | Yes / No |
| - Tumour Mutational Burden |  |  |  |  |  | Yes / No |
| - Microsatellite instability |  |  |  |  |  | Yes / No |
| - Other, namely… |  |  |  |  |  | Yes / No |

Comments:

………………………………………………………………………………………………………………………………………………..………………………………………………………………………………………………………………………………………………..……………………………………………………

1. How would you score the availability and use of the following biomarkers tested for locally advanced or metastatic **glioblastoma** in your country and are they reimbursed?

| **Biomarkers for glioblastoma** | **Never** | **Research** | **Occasionally** | **Usually** | **Always** | **Reimbursed?** |
| --- | --- | --- | --- | --- | --- | --- |
| - IDH mutations |  |  |  |  |  | Yes / No |
| - MGMT methylations |  |  |  |  |  | Yes / No |
| - Tumour Mutational Burden |  |  |  |  |  | Yes / No |
| - Microsatellite instability |  |  |  |  |  | Yes / No |
| - Other, namely… |  |  |  |  |  | Yes / No |

Comments:

………………………………………………………………………………………………………………………………………………..………………………………………………………………………………………………………………………………………………..……………………………………………………

1. Which of the following biomarkers are available in your country for locally advanced or metastatic **Cancer of Unknown Primary (CUP)** and if they are available: how often are they tested and are the tests reimbursed?

| **Biomarkers for CUP** | **Never** | **Research** | **Occasionally** | **Usually** | **Always** | **Reimbursed?** |
| --- | --- | --- | --- | --- | --- | --- |
| - Whole Genome Sequencing |  |  |  |  |  | Yes / No |
| - Next Generation Sequencing *Large panel (>50 genes)* |  |  |  |  |  | Yes / No |
| - Tumour Mutational Burden |  |  |  |  |  | Yes / No |
| - Microsatellite instability |  |  |  |  |  | Yes / No |
| - Other, namely… |  |  |  |  |  | Yes / No |

Comments:

………………………………………………………………………………………………………………………………………………..………………………………………………………………………………………………………………………………………………..……………………………………………………

1. How are the available molecular tests funded?

- All tests are fully reimbursed by the patient’s insurance
- All tests are fully covered by public reimbursement
- (Part of the) tests that are not reimbursed by the patient’s insurance are covered by the hospital
- (Part of the) tests that are not reimbursed by the patient’s insurance are covered by public reimbursement
- (Part of the) tests that are not reimbursed by the patient’s insurance have to be paid by the patient
- The patient has to pay the total price for the tests
- The patient has to pay a fee, the rest is covered by the hospital
- The patient has to pay a fee, the rest is covered by public reimbursement
- The reimbursement differs a lot between hospitals
- Other, namely …

III. BARRIERS

1. Please select for the following possible barriers how often you think they form an obstacle for reimbursement of molecular testing in your country:

|  | **Never** | **Rarely** | **Occasionally** | **Frequently** | **Always** |
| --- | --- | --- | --- | --- | --- |
| The drug that targets the specific biomarker is not available |  |  |  |  |  |
| The drug that targets the specific biomarker is not reimbursed |  |  |  |  |  |
| Lack of evidence that the tests have enough medical implication |  |  |  |  |  |
| The specific mutation is too rare |  |  |  |  |  |
| Other, namely … |  |  |  |  |  |

1. Please select for the following possible barriers how often you think they form an obstacle for performing molecular testing in your country:

|  | **Never** | **Rarely** | **Occasionally** | **Frequently** | **Always** |
| --- | --- | --- | --- | --- | --- |
| The molecular testing procedure is not reimbursed |  |  |  |  |  |
| The drug is not reimbursed |  |  |  |  |  |
| There is no suitable drug available (e.g. off-label) |  |  |  |  |  |
| Difficulty to find a suitable clinical trial |  |  |  |  |  |
| Lack of experience with the procedure |  |  |  |  |  |
| Lack of experience with the interpretation of test results |  |  |  |  |  |
| Lack of evidence for effectiveness of the test |  |  |  |  |  |
| Long turnaround time for test results |  |  |  |  |  |
| Ethical considerations (e.g. incidental results |  |  |  |  |  |
| Other, namely… |  |  |  |  |  |

IV. TESTING

1. When would molecular testing be used in your country?

|  | **Never** | **Research** | **Occasionally** | **Usually** | **Always** |
| --- | --- | --- | --- | --- | --- |
| At initial diagnosis for all patients |  |  |  |  |  |
| At initial diagnosis for high risk patients |  |  |  |  |  |
| At initial diagnosis for metastatic patients |  |  |  |  |  |
| To investigate treatment options in patients who exhausted all standard of care options |  |  |  |  |  |
| To investigate treatment options in rare malignancies |  |  |  |  |  |
| If the malignancy was of unknown origin |  |  |  |  |  |

1. For what reason would molecular testing be used in your country?

|  | **Never** | **Research** | **Occasionally** | **Usually** | **Always** |
| --- | --- | --- | --- | --- | --- |
| For the use of EMA-approved drugs |  |  |  |  |  |
| For off-label use |  |  |  |  |  |
| To provide diagnostic information |  |  |  |  |  |
| To provide prognostic information |  |  |  |  |  |
| For eligibility for clinical trials |  |  |  |  |  |

1. Do you feel that molecular testing is equally available and accessible for all cancer patients in your country?
   - Yes
   - No, there are differences between regions and hospitals
   - Other, ……………..

This is the end of the questionnaire.

#
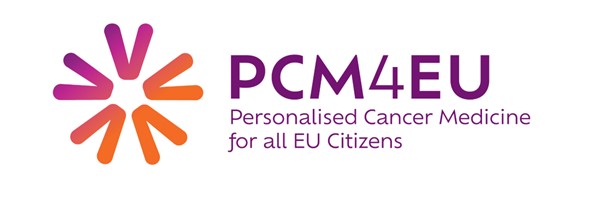
Supplementary Appendix S2. Pathologist questionnaire

*Introduction*

Thank you for taking the time to fill in this questionnaire. The questionnaire is intended for pathologists or molecular biologists who work at a laboratory that performs molecular testing and is affiliated with a DRUP-like clinical trial in their country or part of the formation of a DRUP-like clinical trial, all contributing in the PCM4EU project, and consists of questions regarding the use and reimbursement of molecular testing.

The questionnaire is divided into three topics. The first part contains general questions, the second part includes questions on the availability, use and reimbursement of biomarker testing techniques per tumour type (15 in total), and the third part consists of questions on types of laboratories and prizes.

It will take around 30 minutes to complete the questionnaire. It is possible to close the questionnaire and finish it at a later stage. The answers will be saved.

By checking the box below you agree that your results will be saved and used for research purposes.

- I agree

I. GENERAL

1. Which country do you live in?

- Croatia
- Denmark
- Estonia
- Finland
- France
- Germany
- Hungary
- Italy
- Lithuania
- Norway
- Poland
- Portugal
- Spain
- Sweden
- The Netherlands
- Other, namely …………………….

1. How many laboratories does your country have in total that can perform molecular testing?

…………………………… laboratories

- 1. How many of these laboratories are ISO accredited?

……………………………………… laboratories

- 1. How many of these laboratories are not ISO accredited but participate in at least one External Quality Assessment (EQA)?

……………………………………… laboratories

1. Does your country have one or multiple Molecular Tumour Boards (MTBs)?
   - Yes (go to question 4)
   - No (go to question 5)
2. What type of MTBs are available in your country? (multiple answers possible)
   - National
   - Regional
   - Institutional

II. BIOMARKER TESTING TECHNIQUES AND REIMBURSEMENT

The following questions address the availability and use of biomarker testing techniques per tumour type and if they are reimbursed. Please choose the most suiting option. If there are differences within regions in your country, you can choose multiple options. After each tumour type there is room for additional remarks to specify the regions and elaborate on the options.

1. How would you score the availability and use of the following biomarker testing techniques for locally advanced or metastatic **non-small cell lung cancer (NSCLC)** in your country and are they reimbursed?

| **Biomarker testing techniques used in NSCLC** | **Never** | **Research** | **Occasionally** | **Usually** | **Always** | **Reimbursed?** |
| --- | --- | --- | --- | --- | --- | --- |
| - Immunohistochemistry |  |  |  |  |  | Yes / No |
| - FISH/CISH |  |  |  |  |  | Yes / No |
| - PCR |  |  |  |  |  | Yes / No |
| - Microsatellite instability |  |  |  |  |  | Yes / No |
| - Next Generation Sequencing   *Small panel (<50 genes)* |  |  |  |  |  | Yes / No |
| - Next Generation Sequencing   *Large panel (>50 genes)* |  |  |  |  |  | Yes / No |
| - RNA Sequencing   *Targeted* |  |  |  |  |  | Yes / No |
| - RNA Sequencing - *Full* |  |  |  |  |  | Yes / No |
| - Whole Exome Sequencing |  |  |  |  |  | Yes / No |
| - Whole Genome Sequencing |  |  |  |  |  | Yes / No |
| - Genomic assays |  |  |  |  |  | Yes / No |
| - Liquid biopsies |  |  |  |  |  | Yes / No |
| - Tumour Mutational Burden |  |  |  |  |  | Yes / No |

Comments:

………………………………………………………………………………………………………………………………………………..………………………………………………………………………………………………………………………………………………..……………………………………………………

1. How would you score the availability and use of the following biomarker testing techniques for locally advanced or metastatic **colorectal cancer (CRC)** in your country and are they reimbursed?

| **Biomarker testing techniques used in CRC** | **Never** | **Research** | **Occasionally** | **Usually** | **Always** | **Reimbursed?** |
| --- | --- | --- | --- | --- | --- | --- |
| - Immunohistochemistry |  |  |  |  |  | Yes / No |
| - FISH/CISH |  |  |  |  |  | Yes / No |
| - PCR |  |  |  |  |  | Yes / No |
| - Microsatellite instability |  |  |  |  |  | Yes / No |
| - Next Generation Sequencing   *Small panel (<50 genes)* |  |  |  |  |  | Yes / No |
| - Next Generation Sequencing   *Large panel (>50 genes)* |  |  |  |  |  | Yes / No |
| - RNA Sequencing   *Targeted* |  |  |  |  |  | Yes / No |
| - RNA Sequencing - *Full* |  |  |  |  |  | Yes / No |
| - Whole Exome Sequencing |  |  |  |  |  | Yes / No |
| - Whole Genome Sequencing |  |  |  |  |  | Yes / No |
| - Genomic assays |  |  |  |  |  | Yes / No |
| - Liquid biopsies |  |  |  |  |  | Yes / No |
| - Tumour Mutational Burden |  |  |  |  |  | Yes / No |

Comments:

………………………………………………………………………………………………………………………………………………..………………………………………………………………………………………………………………………………………………..……………………………………………………

1. How would you score the availability and use of the following biomarker testing techniques for locally advanced or metastatic **pancreatic cancer** in your country and are they reimbursed?

| **Biomarker testing techniques used in pancreatic cancer** | **Never** | **Research** | **Occasionally** | **Usually** | **Always** | **Reimbursed?** |
| --- | --- | --- | --- | --- | --- | --- |
| - Immunohistochemistry |  |  |  |  |  | Yes / No |
| - FISH/CISH |  |  |  |  |  | Yes / No |
| - PCR |  |  |  |  |  | Yes / No |
| - Microsatellite instability |  |  |  |  |  | Yes / No |
| - Next Generation Sequencing   *Small panel (<50 genes)* |  |  |  |  |  | Yes / No |
| - Next Generation Sequencing   *Large panel (>50 genes)* |  |  |  |  |  | Yes / No |
| - RNA Sequencing   *Targeted* |  |  |  |  |  | Yes / No |
| - RNA Sequencing - *Full* |  |  |  |  |  | Yes / No |
| - Whole Exome Sequencing |  |  |  |  |  | Yes / No |
| - Whole Genome Sequencing |  |  |  |  |  | Yes / No |
| - Genomic assays |  |  |  |  |  | Yes / No |
| - Liquid biopsies |  |  |  |  |  | Yes / No |
| - Tumour Mutational Burden |  |  |  |  |  | Yes / No |

Comments:

………………………………………………………………………………………………………………………………………………..………………………………………………………………………………………………………………………………………………..……………………………………………………

1. How would you score the availability and use of the following biomarker testing techniques for locally advanced or metastatic **breast cancer** in your country and are they reimbursed?

| **Biomarker testing techniques used in breast cancer** | **Never** | **Research** | **Occasionally** | **Usually** | **Always** | **Reimbursed?** |
| --- | --- | --- | --- | --- | --- | --- |
| - Immunohistochemistry |  |  |  |  |  | Yes / No |
| - FISH/CISH |  |  |  |  |  | Yes / No |
| - PCR |  |  |  |  |  | Yes / No |
| - Microsatellite instability |  |  |  |  |  | Yes / No |
| - Next Generation Sequencing   *Small panel (<50 genes)* |  |  |  |  |  | Yes / No |
| - Next Generation Sequencing   *Large panel (>50 genes)* |  |  |  |  |  | Yes / No |
| - RNA Sequencing   *Targeted* |  |  |  |  |  | Yes / No |
| - RNA Sequencing - *Full* |  |  |  |  |  | Yes / No |
| - Whole Exome Sequencing |  |  |  |  |  | Yes / No |
| - Whole Genome Sequencing |  |  |  |  |  | Yes / No |
| - Genomic assays |  |  |  |  |  | Yes / No |
| - Liquid biopsies |  |  |  |  |  | Yes / No |
| - Tumour Mutational Burden |  |  |  |  |  | Yes / No |

Comments:

………………………………………………………………………………………………………………………………………………..………………………………………………………………………………………………………………………………………………..……………………………………………………

1. How would you score the availability and use of the following biomarker testing techniques for locally advanced or metastatic **prostate cancer** in your country and are they reimbursed?

| **Biomarker testing techniques used in prostate cancer** | **Never** | **Research** | **Occasionally** | **Usually** | **Always** | **Reimbursed?** |
| --- | --- | --- | --- | --- | --- | --- |
| - Immunohistochemistry |  |  |  |  |  | Yes / No |
| - FISH/CISH |  |  |  |  |  | Yes / No |
| - PCR |  |  |  |  |  | Yes / No |
| - Microsatellite instability |  |  |  |  |  | Yes / No |
| - Next Generation Sequencing   *Small panel (<50 genes)* |  |  |  |  |  | Yes / No |
| - Next Generation Sequencing   *Large panel (>50 genes)* |  |  |  |  |  | Yes / No |
| - RNA Sequencing   *Targeted* |  |  |  |  |  | Yes / No |
| - RNA Sequencing - *Full* |  |  |  |  |  | Yes / No |
| - Whole Exome Sequencing |  |  |  |  |  | Yes / No |
| - Whole Genome Sequencing |  |  |  |  |  | Yes / No |
| - Genomic assays |  |  |  |  |  | Yes / No |
| - Liquid biopsies |  |  |  |  |  | Yes / No |
| - Tumour Mutational Burden |  |  |  |  |  | Yes / No |

Comments:

………………………………………………………………………………………………………………………………………………..………………………………………………………………………………………………………………………………………………..……………………………………………………

1. How would you score the availability and use of the following biomarker testing techniques for locally advanced or metastatic **melanoma** in your country and are they reimbursed?

| **Biomarker testing techniques used in melanoma** | **Never** | **Research** | **Occasionally** | **Usually** | **Always** | **Reimbursed?** |
| --- | --- | --- | --- | --- | --- | --- |
| - Immunohistochemistry |  |  |  |  |  | Yes / No |
| - FISH/CISH |  |  |  |  |  | Yes / No |
| - PCR |  |  |  |  |  | Yes / No |
| - Microsatellite instability |  |  |  |  |  | Yes / No |
| - Next Generation Sequencing   *Small panel (<50 genes)* |  |  |  |  |  | Yes / No |
| - Next Generation Sequencing   *Large panel (>50 genes)* |  |  |  |  |  | Yes / No |
| - RNA Sequencing   *Targeted* |  |  |  |  |  | Yes / No |
| - RNA Sequencing - *Full* |  |  |  |  |  | Yes / No |
| - Whole Exome Sequencing |  |  |  |  |  | Yes / No |
| - Whole Genome Sequencing |  |  |  |  |  | Yes / No |
| - Genomic assays |  |  |  |  |  | Yes / No |
| - Liquid biopsies |  |  |  |  |  | Yes / No |
| - Tumour Mutational Burden |  |  |  |  |  | Yes / No |

Comments:

………………………………………………………………………………………………………………………………………………..………………………………………………………………………………………………………………………………………………..……………………………………………………

1. How would you score the availability and use of the following biomarker testing techniques for locally advanced or metastatic **ovarian cancer** in your country and are they reimbursed?

| **Biomarker testing techniques used in ovarian cancer** | **Never** | **Research** | **Occasionally** | **Usually** | **Always** | **Reimbursed?** |
| --- | --- | --- | --- | --- | --- | --- |
| - Immunohistochemistry |  |  |  |  |  | Yes / No |
| - FISH/CISH |  |  |  |  |  | Yes / No |
| - PCR |  |  |  |  |  | Yes / No |
| - Microsatellite instability |  |  |  |  |  | Yes / No |
| - Next Generation Sequencing   *Small panel (<50 genes)* |  |  |  |  |  | Yes / No |
| - Next Generation Sequencing   *Large panel (>50 genes)* |  |  |  |  |  | Yes / No |
| - RNA Sequencing   *Targeted* |  |  |  |  |  | Yes / No |
| - RNA Sequencing - *Full* |  |  |  |  |  | Yes / No |
| - Whole Exome Sequencing |  |  |  |  |  | Yes / No |
| - Whole Genome Sequencing |  |  |  |  |  | Yes / No |
| - Genomic assays |  |  |  |  |  | Yes / No |
| - Liquid biopsies |  |  |  |  |  | Yes / No |
| - Tumour Mutational Burden |  |  |  |  |  | Yes / No |

Comments:

………………………………………………………………………………………………………………………………………………..………………………………………………………………………………………………………………………………………………..……………………………………………………

1. How would you score the availability and use of the following biomarker testing techniques for locally advanced or metastatic **endometrial cancer** in your country and are they reimbursed?

| **Biomarker testing techniques used in endometrial cancer** | **Never** | **Research** | **Occasionally** | **Usually** | **Always** | **Reimbursed?** |
| --- | --- | --- | --- | --- | --- | --- |
| - Immunohistochemistry |  |  |  |  |  | Yes / No |
| - FISH/CISH |  |  |  |  |  | Yes / No |
| - PCR |  |  |  |  |  | Yes / No |
| - Microsatellite instability |  |  |  |  |  | Yes / No |
| - Next Generation Sequencing   *Small panel (<50 genes)* |  |  |  |  |  | Yes / No |
| - Next Generation Sequencing   *Large panel (>50 genes)* |  |  |  |  |  | Yes / No |
| - RNA Sequencing   *Targeted* |  |  |  |  |  | Yes / No |
| - RNA Sequencing - *Full* |  |  |  |  |  | Yes / No |
| - Whole Exome Sequencing |  |  |  |  |  | Yes / No |
| - Whole Genome Sequencing |  |  |  |  |  | Yes / No |
| - Genomic assays |  |  |  |  |  | Yes / No |
| - Liquid biopsies |  |  |  |  |  | Yes / No |
| - Tumour Mutational Burden |  |  |  |  |  | Yes / No |

Comments:

………………………………………………………………………………………………………………………………………………..………………………………………………………………………………………………………………………………………………..……………………………………………………

1. How would you score the availability and use of the following biomarker testing techniques for locally advanced or metastatic **cervical cancer** in your country and are they reimbursed?

| **Biomarker testing techniques used in cervical cancer** | **Never** | **Research** | **Occasionally** | **Usually** | **Always** | **Reimbursed?** |
| --- | --- | --- | --- | --- | --- | --- |
| - Immunohistochemistry |  |  |  |  |  | Yes / No |
| - FISH/CISH |  |  |  |  |  | Yes / No |
| - PCR |  |  |  |  |  | Yes / No |
| - Microsatellite instability |  |  |  |  |  | Yes / No |
| - Next Generation Sequencing   *Small panel (<50 genes)* |  |  |  |  |  | Yes / No |
| - Next Generation Sequencing   *Large panel (>50 genes)* |  |  |  |  |  | Yes / No |
| - RNA Sequencing   *Targeted* |  |  |  |  |  | Yes / No |
| - RNA Sequencing - *Full* |  |  |  |  |  | Yes / No |
| - Whole Exome Sequencing |  |  |  |  |  | Yes / No |
| - Whole Genome Sequencing |  |  |  |  |  | Yes / No |
| - Genomic assays |  |  |  |  |  | Yes / No |
| - Liquid biopsies |  |  |  |  |  | Yes / No |
| - Tumour Mutational Burden |  |  |  |  |  | Yes / No |

Comments:

………………………………………………………………………………………………………………………………………………..………………………………………………………………………………………………………………………………………………..……………………………………………………

1. How would you score the availability and use of the following biomarker testing techniques for locally advanced or metastatic **renal cell/urothelial cell cancer** in your country and are they reimbursed?

| **Biomarker testing techniques used in renal cell/urothelial cell cancer** | **Never** | **Research** | **Occasionally** | **Usually** | **Always** | **Reimbursed?** |
| --- | --- | --- | --- | --- | --- | --- |
| - Immunohistochemistry |  |  |  |  |  | Yes / No |
| - FISH/CISH |  |  |  |  |  | Yes / No |
| - PCR |  |  |  |  |  | Yes / No |
| - Microsatellite instability |  |  |  |  |  | Yes / No |
| - Next Generation Sequencing   *Small panel (<50 genes)* |  |  |  |  |  | Yes / No |
| - Next Generation Sequencing   *Large panel (>50 genes)* |  |  |  |  |  | Yes / No |
| - RNA Sequencing   *Targeted* |  |  |  |  |  | Yes / No |
| - RNA Sequencing - *Full* |  |  |  |  |  | Yes / No |
| - Whole Exome Sequencing |  |  |  |  |  | Yes / No |
| - Whole Genome Sequencing |  |  |  |  |  | Yes / No |
| - Genomic assays |  |  |  |  |  | Yes / No |
| - Liquid biopsies |  |  |  |  |  | Yes / No |
| - Tumour Mutational Burden |  |  |  |  |  | Yes / No |

Comments:

………………………………………………………………………………………………………………………………………………..………………………………………………………………………………………………………………………………………………..……………………………………………………

1. How would you score the availability and use of the following biomarker testing techniques for locally advanced or metastatic **biliary tract cancer** in your country and are they reimbursed?

| **Biomarker testing techniques used in biliary tract cancer** | **Never** | **Research** | **Occasionally** | **Usually** | **Always** | **Reimbursed?** |
| --- | --- | --- | --- | --- | --- | --- |
| - Immunohistochemistry |  |  |  |  |  | Yes / No |
| - FISH/CISH |  |  |  |  |  | Yes / No |
| - PCR |  |  |  |  |  | Yes / No |
| - Microsatellite instability |  |  |  |  |  | Yes / No |
| - Next Generation Sequencing   *Small panel (<50 genes)* |  |  |  |  |  | Yes / No |
| - Next Generation Sequencing   *Large panel (>50 genes)* |  |  |  |  |  | Yes / No |
| - RNA Sequencing   *Targeted* |  |  |  |  |  | Yes / No |
| - RNA Sequencing - *Full* |  |  |  |  |  | Yes / No |
| - Whole Exome Sequencing |  |  |  |  |  | Yes / No |
| - Whole Genome Sequencing |  |  |  |  |  | Yes / No |
| - Genomic assays |  |  |  |  |  | Yes / No |
| - Liquid biopsies |  |  |  |  |  | Yes / No |
| - Tumour Mutational Burden |  |  |  |  |  | Yes / No |

Comments:

………………………………………………………………………………………………………………………………………………..………………………………………………………………………………………………………………………………………………..……………………………………………………

1. How would you score the availability and use of the following biomarker testing techniques for locally advanced or metastatic **gastrointestinal stromal tumour (GIST)** in your country and are they reimbursed?

| **Biomarker testing techniques used in GIST** | **Never** | **Research** | **Occasionally** | **Usually** | **Always** | **Reimbursed?** |
| --- | --- | --- | --- | --- | --- | --- |
| - Immunohistochemistry |  |  |  |  |  | Yes / No |
| - FISH/CISH |  |  |  |  |  | Yes / No |
| - PCR |  |  |  |  |  | Yes / No |
| - Microsatellite instability |  |  |  |  |  | Yes / No |
| - Next Generation Sequencing   *Small panel (<50 genes)* |  |  |  |  |  | Yes / No |
| - Next Generation Sequencing   *Large panel (>50 genes)* |  |  |  |  |  | Yes / No |
| - RNA Sequencing   *Targeted* |  |  |  |  |  | Yes / No |
| - RNA Sequencing - *Full* |  |  |  |  |  | Yes / No |
| - Whole Exome Sequencing |  |  |  |  |  | Yes / No |
| - Whole Genome Sequencing |  |  |  |  |  | Yes / No |
| - Genomic assays |  |  |  |  |  | Yes / No |
| - Liquid biopsies |  |  |  |  |  | Yes / No |
| - Tumour Mutational Burden |  |  |  |  |  | Yes / No |

Comments:

………………………………………………………………………………………………………………………………………………..………………………………………………………………………………………………………………………………………………..……………………………………………………

1. How would you score the availability and use of the following biomarker testing techniques for locally advanced or metastatic **thyroid cancer** in your country and are they reimbursed?

| **Biomarker testing techniques used in thyroid cancer** | **Never** | **Research** | **Occasionally** | **Usually** | **Always** | **Reimbursed?** |
| --- | --- | --- | --- | --- | --- | --- |
| - Immunohistochemistry |  |  |  |  |  | Yes / No |
| - FISH/CISH |  |  |  |  |  | Yes / No |
| - PCR |  |  |  |  |  | Yes / No |
| - Microsatellite instability |  |  |  |  |  | Yes / No |
| - Next Generation Sequencing   *Small panel (<50 genes)* |  |  |  |  |  | Yes / No |
| - Next Generation Sequencing   *Large panel (>50 genes)* |  |  |  |  |  | Yes / No |
| - RNA Sequencing   *Targeted* |  |  |  |  |  | Yes / No |
| - RNA Sequencing - *Full* |  |  |  |  |  | Yes / No |
| - Whole Exome Sequencing |  |  |  |  |  | Yes / No |
| - Whole Genome Sequencing |  |  |  |  |  | Yes / No |
| - Genomic assays |  |  |  |  |  | Yes / No |
| - Liquid biopsies |  |  |  |  |  | Yes / No |
| - Tumour Mutational Burden |  |  |  |  |  | Yes / No |

Comments:

………………………………………………………………………………………………………………………………………………..………………………………………………………………………………………………………………………………………………..……………………………………………………

1. How would you score the availability and use of the following biomarker testing techniques for **glioblastoma** in your country and are they reimbursed?

| **Biomarker testing techniques used in glioblastoma** | **Never** | **Research** | **Occasionally** | **Usually** | **Always** | **Reimbursed?** |
| --- | --- | --- | --- | --- | --- | --- |
| - Immunohistochemistry |  |  |  |  |  | Yes / No |
| - FISH/CISH |  |  |  |  |  | Yes / No |
| - PCR |  |  |  |  |  | Yes / No |
| - Microsatellite instability |  |  |  |  |  | Yes / No |
| - Next Generation Sequencing   *Small panel (<50 genes)* |  |  |  |  |  | Yes / No |
| - Next Generation Sequencing   *Large panel (>50 genes)* |  |  |  |  |  | Yes / No |
| - RNA Sequencing   *Targeted* |  |  |  |  |  | Yes / No |
| - RNA Sequencing - *Full* |  |  |  |  |  | Yes / No |
| - Whole Exome Sequencing |  |  |  |  |  | Yes / No |
| - Whole Genome Sequencing |  |  |  |  |  | Yes / No |
| - Genomic assays |  |  |  |  |  | Yes / No |
| - Liquid biopsies |  |  |  |  |  | Yes / No |
| - Tumour Mutational Burden |  |  |  |  |  | Yes / No |

Comments:

………………………………………………………………………………………………………………………………………………..………………………………………………………………………………………………………………………………………………..……………………………………………………

1. How would you score the availability and use of the following biomarker testing techniques for **cancer of unknown primary (CUP)** in your country and are they reimbursed?

| **Biomarker testing techniques used in CUP** | **Never** | **Research** | **Occasionally** | **Usually** | **Always** | **Reimbursed?** |
| --- | --- | --- | --- | --- | --- | --- |
| - Immunohistochemistry |  |  |  |  |  | Yes / No |
| - FISH/CISH |  |  |  |  |  | Yes / No |
| - PCR |  |  |  |  |  | Yes / No |
| - Microsatellite instability |  |  |  |  |  | Yes / No |
| - Next Generation Sequencing   *Small panel (<50 genes)* |  |  |  |  |  | Yes / No |
| - Next Generation Sequencing   *Large panel (>50 genes)* |  |  |  |  |  | Yes / No |
| - RNA Sequencing   *Targeted* |  |  |  |  |  | Yes / No |
| - RNA Sequencing - *Full* |  |  |  |  |  | Yes / No |
| - Whole Exome Sequencing |  |  |  |  |  | Yes / No |
| - Whole Genome Sequencing |  |  |  |  |  | Yes / No |
| - Genomic assays |  |  |  |  |  | Yes / No |
| - Liquid biopsies |  |  |  |  |  | Yes / No |
| - Tumour Mutational Burden |  |  |  |  |  | Yes / No |

Comments:

………………………………………………………………………………………………………………………………………………..………………………………………………………………………………………………………………………………………………..……………………………………………………

1. How are all the available tests funded?

- All tests are fully reimbursed by the patient’s insurance
- All tests are fully covered by public reimbursement
- (Part of the) tests that are not reimbursed by the patient’s insurance are covered by the hospital
- (Part of the) tests that are not reimbursed by the patient’s insurance are covered by public reimbursement
- (Part of the) tests that are not reimbursed by the patient’s insurance have to be paid by the patient
- The patient has to pay the total price for the tests
- The patient has to pay a fee, the rest is covered by the hospital
- The patient has to pay a fee, the rest is covered by public reimbursement
- The reimbursement differs a lot between laboratories
- Other, namely …………………..

III. LABORATORY AND PRIZING

1. In what type of laboratories are the biomarker testing techniques performed?

| **Biomarker testing techniques** | **Public laboratory** | **Both public and private laboratory** | **Private laboratory** | **Not applicable** |
| --- | --- | --- | --- | --- |
| - Immunohistochemistry |  |  |  |  |
| - FISH/CISH |  |  |  |  |
| - PCR |  |  |  |  |
| - Microsatellite instability |  |  |  |  |
| - Next Generation Sequencing   *Small panel (<50 genes)* |  |  |  |  |
| - Next Generation Sequencing   *Large panel (>50 genes)* |  |  |  |  |
| - RNA Sequencing   *Targeted* |  |  |  |  |
| - RNA Sequencing - *Full* |  |  |  |  |
| - Whole Exome Sequencing |  |  |  |  |
| - Whole Genome Sequencing |  |  |  |  |
| - Genomic assays |  |  |  |  |
| - Liquid biopsies |  |  |  |  |
| - Tumour Mutational Burden |  |  |  |  |

1. What is the price of the different biomarker testing techniques in your country?

| **Biomarker testing techniques** | **Price per test (€)** | **Not applicable** |
| --- | --- | --- |
| - Immunohistochemistry |  |  |
| - FISH/CISH |  |  |
| - PCR |  |  |
| - Microsatellite instability |  |  |
| - Next Generation Sequencing   *Small panel (<50 genes)* |  |  |
| - Next Generation Sequencing   *Large panel (>50 genes)* |  |  |
| - RNA Sequencing   *Targeted* |  |  |
| - RNA Sequencing - *Full* |  |  |
| - Whole Exome Sequencing |  |  |
| - Whole Genome Sequencing |  |  |
| - Genomic assays |  |  |
| - Liquid biopsies |  |  |
| - Tumour Mutational Burden |  |  |

This is the end of the questionnaire.

#
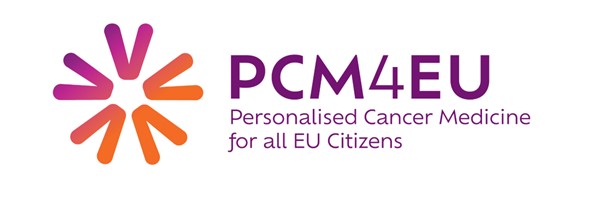
Supplementary Appendix S3. Patient questionnaire

**Recruitment text**

Molecular testing questionnaire

The PCM4EU project (Personalized Cancer Medicine for all EU Citizens) is a large project funded by the European Union. We are looking for adult patients with advanced or metastatic cancer to fill in a questionnaire about molecular testing. With 10 minutes of your time you can help us understand the access to molecular testing in your country. The goal of the PCM4EU project is to give all patients in Europe equal access to molecular testing. Access to testing can potentially lead to access to additional treatment options. Mapping out the current situation helps with that.

Your participation would be greatly appreciated! By clicking on the following link you will go to the online questionnaire: [link]

*Some extra information*

There have been many developments within precision cancer medicine in recent years. Treatments like immunotherapy or targeted therapy offer new possibilities for patients. These treatments target the tumour based on specific characteristics or mutations. Molecular testing can identify those characteristics by testing the DNA of the tumour. The PCM4EU project aims to provide equal access to precision cancer medicine for all European citizens. The goal is to improve survival and quality of life for these patients. More information about the PCM4EU project can be found on the website: <https://www.matrix-fkb.no/en/pcm4eu/home>

**Introductory text**

*Introduction*

Thank you for taking the time to fill in this questionnaire. The questionnaire is intended for adult patients with locally advanced or metastatic cancer. The questionnaire consists of questions about ‘molecular testing’, which may also be called ‘biomarker testing’ or ‘genetic testing’ in clinical practice. With these tests the genetic profile of a tumour is analysed to identify certain mutations that can be targeted for therapy. In this questionnaire the term ‘molecular testing’ will be used.

The questionnaire is divided into four topics. The first part starts with some general questions. The second part includes questions on the molecular testing procedure. The third part contains some questions to assess your knowledge on the topic. The fourth part consists of questions about the test results.

You will have to complete the questionnaire in total straight away. It is not possible to stop halfway and continue later where you left off. Completing the questionnaire will take approximately 10 minutes.

Some questions may not be relevant in your country due to local, legal or ethical rules. When answering the questions, please choose the most relevant option.

By checking the box below you agree that your results will be saved and used for research purposes. All questions are anonymous and cannot be traced back to a specific person.

- I agree

I. DEMOGRAPHICS

1. What type of cancer do you have?
   - Bladder cancer
   - Breast cancer
   - Central nervous system cancer (brain and spinal cord)
   - Cervical cancer
   - Colorectal cancer
   - Gallbladder cancer
   - Kidney cancer
   - Lung cancer
   - Melanoma
   - Oesophageal cancer
   - Oral and oropharyngeal cancer
   - Ovarian cancer
   - Pancreatic cancer
   - Prostate cancer
   - Sarcoma
   - Testicular cancer
   - Thyroid cancer
   - Uterine cancer
   - Other, namely ……………
2. Is your cancer in a locally advanced stage or metastasized?
   - Yes
   - No
   - I do not know

*If you do not have locally advanced or metastatic cancer, this is the end of the questionnaire.*

1. Which country do you live in?
   - Croatia
   - Denmark
   - Estonia
   - Finland
   - France
   - Germany
   - Hungary
   - Italy
   - Lithuania
   - Norway
   - Poland
   - Portugal
   - Spain
   - Sweden
   - The Netherlands
   - Other, namely …………………….
2. What is your age?
   - 18-25 years
   - 26-40 years
   - 41-60 years
   - 61-75 years
   - >75 years
3. What is your highest level of education?
   - Primary education (primary school)
   - Secondary education (high school)
   - Vocational education
   - Short higher education (up to 3 years)
   - Long higher education (4 years or more)
   - Other, namely ………..
4. What type of hospital are you or have you been treated in?
   - University hospital or cancer centre
   - Regional hospital (a smaller hospital with patient care as primary focus)
   - Outpatient clinic (a privately operated healthcare facility)
   - I do not know
5. How far do you live from the nearest university hospital or cancer centre?
   - Less than 10 kilometres
   - 10-30 kilometres
   - 30-50 kilometres
   - 50-100 kilometres
   - >100 kilometres

II. MOLECULAR TESTING PROCEDURE

1. Did you undergo molecular testing of the DNA of the tumour before or anywhere during your anti-cancer treatment?
   - Yes
   - No
   - I do not know

*If you did not undergo molecular testing, this is the end of the questionnaire.*

1. When did you undergo molecular testing? *(more answers possible)*
   - After the initial diagnosis, before the start of any anti-cancer treatment
   - In between two types of treatment
   - After all standard treatment options were exhausted
   - Before inclusion in a clinical trial
   - I underwent molecular testing, but I do not know when
2. Who ordered the molecular testing?
   - My doctor ordered the molecular testing
   - I found a facility and ordered molecular testing myself
   - I do not know
   - Other, namely …………………………
3. Has your doctor explained how the molecular test results would influence the treatment options?
   - Yes
   - No
   - I do not know
4. Did you undergo genetic counselling before starting the molecular testing procedure?
   - Yes
   - No
   - I do not know
5. In what ways were you informed about the molecular testing procedure? *(more answers possible)*
   - My doctor (or other health care professional) explained the procedure
   - I received an information folder from my doctor/nurse
   - I searched for information online
   - I did not receive any information on the molecular testing procedure
   - I do not remember how I received the information
   - Other, namely ……………….
6. How well were you informed about the molecular testing procedure?

Please rate on a scale from 1 to 5.
1 = not informed at all
2 = not well informed
3 = moderately informed
4 = well informed
5 = very well informed

1. In what way was the molecular testing paid for?
   - I had to pay for the testing myself
   - It was covered by my insurance
   - It was covered by the hospital
   - It was covered by public reimbursement
   - It was partially reimbursed, I had to pay a contribution
   - I did not have to pay anything, but I do not know how it was reimbursed
   - I do not remember
   - Other, namely …………………..

III. KNOWLEDGE ON MOLECULAR TESTING

1. Where did you learn about the term ‘molecular testing’? *(more answers possible)*
   - My doctor (or other health care professional) told me about it
   - Via my cancer patient organisation
   - Via online research I conducted myself
   - Via social media
   - I just learned about this term during this questionnaire
   - Other, namely …………………………..
2. Which of the following statements is true?
   - If a mutation happens in a cell this will always result in the development of cancer
   - Genes consist of parts of DNA
   - Cancer is always aggressive
   - All of the above
   - I do not know
3. The majority of cancer is hereditary.
   - True
   - False
   - I do not know
4. Testing for mutations that are inherited within families (germline mutations) happens via:
   - A blood sample or cheek swab
   - Tumour sample
   - It can be tested in both ways
   - I do not know
5. Molecular testing of a tumour can be used for: *(more answers possible)*
   - Identification of a cancer type
   - Prediction of someone’s risk to develop cancer
   - Prediction if someone will respond to a particular treatment
   - Prediction of disease outcome
   - I do not know

IV. TEST RESULTS

1. How long did it take before you received the molecular testing results after collection of the test sample (turnaround time)?
   - Less than 2 weeks
   - Between 2 and 4 weeks
   - More than 4 weeks
   - I do not know
2. Would you want to know if any of the found mutations turned out to be hereditary?
   - Yes
   - No
   - I do not know
3. Were any of the mutations that were found during molecular testing hereditary?
   - Yes, there were mutations found that were hereditary
   - No, there were mutations found, but they were not hereditary
   - There were no mutations found
   - I do not know
4. Did you receive treatment based on the molecular testing results?
   - Yes, I was admitted in a clinical trial and received specific treatment
   - Yes, I received specific treatment, but this was not in a clinical trial
   - No, I did not receive treatment based on the molecular testing results
   - I do not know
5. Did you have to go to another hospital for treatment after the results of the molecular testing came in?
   - Yes, the treatment I was able to get after molecular testing was performed, was not available at my hospital
   - No, I did not have to go to another hospital for the treatment
   - I did not receive treatment based on the molecular testing
   - I do not know
6. Were you satisfied with the information provided by your doctor (or other health care professional) about the test results and what the consequences were for your treatment options? Please rate on a scale from 1 to 5.
   1 = very dissatisfied
   2 = slightly dissatisfied
   3 = reasonably satisfied
   4 = quite satisfied
   5 = very satisfied
7. If you would be able to get treatment in a clinical trial, but that trial would only be available abroad, would you go to that country to get treatment?
   - Yes, I would go abroad, even if I have to pay for it myself
   - Yes, I would go abroad, but only if everything is covered by insurance or any kind of funding
   - Yes, I would go abroad, but only if the country is nearby
   - No, I would not travel abroad for treatment
   - Other, namely ……………………
8. Would you be okay with your medical information being shared anonymously with other hospitals **in your country** for research purposes?
   - Yes, I would be fine with my medical information being shared for research purposes
   - I only want my medical information to be shared if I could get treatment, not for research purposes
   - No, I don’t want my medical information to be shared
   - Other, namely ………………
9. Would you be okay with your medical information being shared anonymously with other hospitals **abroad** for research purposes?
   - Yes, I would be fine with my medical information being shared for research purposes
   - I only want my medical information to be shared if I could get treatment, not for research purposes
   - No, I don’t want my medical information to be shared
   - Other, namely ………………
10. Looking at your overall experience with molecular testing, what could be changed to improve your experience? *(multiple answers possible)*
    - I would change nothing, I was very satisfied
    - Provide more understandable information on the topic
    - Provide pre- and post-test counselling
    - Improve turnaround time and provide updates if any delays occur
    - Reimbursement of all of the testing
    - I do not know
    - Other, namely …………………………………………….

This is the end of the questionnaire.
